# Inter‐Nanoparticle FRET for Biosensing: Photophysics Versus Size

**DOI:** 10.1002/anie.202510801

**Published:** 2025-08-14

**Authors:** Eduard Madirov, Clara Catros, Niko Hildebrandt, Chloé Grazon

**Affiliations:** ^1^ Department of Engineering Physics McMaster University Hamilton ON M8S 4K1 Canada; ^2^ Univ. Bordeaux, CNRS, Bordeaux INP, ISM UMR 5255 Talence F‐33400 France

**Keywords:** Dyes, Fluorescence, Polymer nanoparticles, Quantum dots, Upconversion nanoparticles

## Abstract

Förster resonance energy transfer (FRET) enables the quantification of nanoscale distances and biomolecular interactions. Luminescent nanoparticles (NPs) are frequently combined with fluorescent dyes or proteins in FRET. However, their use in inter‐NP FRET with luminescent NPs as both donor and acceptor remains less common due to the inherent size constraints that can limit FRET efficiencies. This review critically examines the early advances and current state‐of‐the‐art of inter‐NP FRET with a focus on the most commonly used NPs, namely quantum dots (QDs), upconversion nanoparticles (UCNPs), and fluorescent organic nanoparticles (FONs). We show how NP sizes, surface shells, and coatings increase FRET‐distances; propose how these drawbacks can be overcome or outcompeted by the unique photophysical properties of the NPs; and discuss representative examples of inter‐NP FRET for biosensing. High luminescence brightness, outstanding photostability, near‐infrared excitation and emission, spectral and temporal multiplexing, and large surfaces for multifunctional bioconjugation are only some of the features that make luminescent NPs very attractive for biosensing, and FRET efficiencies up to 90% have demonstrated their potential for successful translation into bioanalytical applications.

## Graphical abstract

Förster resonance energy transfer (FRET) systems with luminescent nanoparticles (NPs) as donors and acceptors (inter‐NP FRET) combine unique photophysical properties with large sizes. This review discusses the influence of NP type, architecture, surface chemistry, and optical properties on FRET and the pros and cons of inter‐NP FRET for bioanalytical applications.

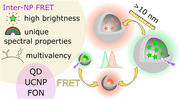

## 1 Introduction

Förster resonance energy transfer (FRET)^[^ ^1^, ^2^ ^]^ is a powerful technique to study molecular interactions, structural dynamics, and biosensing.^[^ ^3^, ^4^, ^5^, ^6^ ^]^ FRET is a non‐radiative energy transfer between a luminescent donor and an absorbing acceptor via dipole–dipole interaction that happens within the near‐field of the donor with an efficiency i.e., proportional to the inverse sixth power of the donor–acceptor distance. Typical FRET distances range from 1 to 10 nm, which correspond to the size of biomacromolecules such as antibodies, proteins, and DNA. FRET can be used as a molecular ruler for distance measurements^[^ ^7^, ^8^, ^9^ ^]^ or for the development of biosensors, which has been widely explored since the 1990s.^[^ ^10^ ^]^ Arguably, most FRET probes are still based on organic dyes^[^ ^11^, ^12^, ^13^, ^14^ ^]^ and fluorescent proteins.^[^ ^15^, ^16^ ^]^ However, there are many materials available for designing FRET probes,^[^ ^17^ ^]^ including luminescent nanoparticles (NPs), which can provide advantages over traditional fluorophores, such as high brightness and photostability and a large surface for the attachments of biomolecules and FRET partners (donors or acceptors).^[^ ^18^ ^]^

While luminescent NPs have been widely used as FRET donors or acceptors, FRET between luminescent NPs (inter‐NP FRET) has been much more limited. Despite the photophysical and photochemical advantages of luminescent NPs, their relatively large sizes on the FRET scale make the design of efficient inter‐NP FRET systems challenging. Considering that FRET can function over distances up to approximately 10 nm (or in some cases even up to ca. 20 nm),^[^ ^19^ ^]^ NPs with sizes above 10 nm can significantly limit the FRET efficiency. Therefore, most NP‐based FRET systems consist of one luminescent NP (donor or acceptor) combined with a molecular FRET partner (acceptor or donor), such as lanthanide complexes and quantum dots (QDs) or upconversion NPs (UCNPs) and dyes.^[^ ^18^, ^19^, ^20^ ^]^ Plasmonic NPs (e.g., gold NPs) are also frequently used as quenching energy transfer acceptors, in which case the usual terminology is nanosurface energy transfer (NSET) with an inverse fourth power distance dependence and a slightly longer donor–acceptor distance range.^[^ ^21^, ^22^ ^]^ Thus, FRET systems that use luminescent NPs as both donors and acceptors are much less common because the advantageous photophysical properties need to be weighed against large donor–acceptor distances. The sizes of coatings and biomolecules on the NP surface can further extend the donor–acceptor distance. Owing to these distance and size issues, inter‐NP FRET is not straightforward, and photophysics alone is not sufficient to design a high‐performance FRET system.

Despite the, often, long donor–acceptor distances, inter‐NP FRET is an intriguing topic because very unique photophysical properties are implemented on both sides of the FRET system. With careful design optimization, the photophysical benefits have the potential to outperform the distance disadvantages, and very special FRET systems can be developed. Compared to molecular FRET pairs, the higher brightness, better chemical and physical stability, and increased versatility in excitation and emission wavelengths of luminescent NPs have the potential for lower detection limits, higher sensitivities, higher multiplexing capability, and sensing in challenging biological environments, all of which are highly desirable features for biosensing and bioimaging. Here, we review the state‐of‐the art of inter‐NP FRET, discuss their pros and cons, and provide a critical view on their application in biosensing. Owing to their predominance in the field, we focus our discussion on QDs, UCNPs, and fluorescent organic NPs (FONs), which include semi‐conducting polymer nanoparticles (Pdots),^[^ ^23^ ^]^ dye‐doped polymeric NPs (PNPs),^[^ ^24^ ^]^ and carbon dots (Cdots).^[^ ^25^ ^]^ We exclusively focus on pairs of luminescent NPs, and thus, NSET systems that use plasmonic NPs as acceptors are not reviewed. Other luminescent NPs (e.g., dye‐doped silica NPs or carbon nanotubes) have been extensively used in FRET‐based biosensors but not in inter‐NP FRET systems. After a brief introduction of the FRET principles that apply to luminescent NPs and a short overview of general FRET biosensing applications with QDs, UCNPs, and FONs, we discuss representative examples of QD‐QD, UCNP‐QD, FON–FON, and hybrid organic/inorganic combinations for FRET. Finally, we provide an outlook on challenges that must be overcome to advance beyond the proof‐of‐concept stage, including more compact NP designs, higher brightness per volume, multiplexing capabilities, and the application of organic/inorganic hybrid systems.

## 2 NP FRET in a Nutshell

The basic rules of FRET apply to luminescent NPs as to any other FRET material.^[^ ^3^ ^]^ Important FRET parameters to consider are the donor–acceptor distance (*R*_DA_), the FRET efficiency (*E*_FRET_), the Förster distance (*R*_0_; equals *R*_DA_ at *E*_FRET_ = 0.5), and the spectral overlap integral between donor emission and acceptor absorption (*J*). Because FRET systems with luminescent NPs can have multiple absorbers and emitters, the number of donors per acceptor (*m*) and the number of acceptors per donor (*n*) must also be taken into account.^[^ ^6^, ^26^, ^27^ ^]^ *E*_FRET_ only depends on the number of acceptors, and for the simplest case of equal distances between all donor–acceptor pairs it can be calculated by Equation (1), which depends on Equations (2) and (3).^[^ ^6^, ^26^, ^27^ ^]^

(1) $$E_{\mathrm{FRET}}=\frac{n{R_{0}}^{6}}{n{R_{0}}^{6}+{R_{\mathrm{DA}}}^{6}}$$

(2) $$R_{0}=0.021{\left[\kappa^{2}n_{r}^{-4}\Phi_{D}J\right]}^{1/6}\left(\mathrm{in}\;\mathrm{nm}\right)$$

(3) $$J=\int {\bar{I}}_{D}\left(\lambda \right)\varepsilon_{A}\left(\lambda \;\right)\lambda^{4}d\lambda$$

In these equations, κ^2^ presents the orientation between the donor and acceptor transition dipole moments, *n*_r_ is the refractive index of the surrounding medium, Φ is the luminescence quantum yield, $\bar{I}$ is the normalized emission intensity, ε is the molar extinction coefficient, λ is the wavelength, and subscripts D and A refer to the donor and acceptor, respectively. The number of donors per acceptor does not influence the FRET efficiency, but it influences the probability of acceptor‐sensitization via FRET (*P*_A_), which can be expressed via Equation (4)^[^ ^6^, ^26^, ^27^ ^]^:

(4) $$P_{A}=1-{\left(1-E_{\mathrm{FRET}}\right)}^{m}=1-{\left(\frac{{R_{\mathrm{DA}}}^{6}}{{R_{0}}^{6}+{R_{\mathrm{DA}}}^{6}}\right)}^{m}$$

Both *E*_FRET_ and *P*_A_ are important for FRET biosensing with luminescent NPs because one would ideally like to design a ratiometric sensing system (detecting both quenched donor and sensitized acceptor emission) with a large absorption cross section, a sufficiently high FRET efficiency, and a significantly sensitized acceptor. *E*_FRET_ and *P*_A_ depend on both the photophysical properties (*R*_0_) and the distance (*R*_DA_) between donor and acceptor.

### 2.1 Photophysics

**Figure 1:**
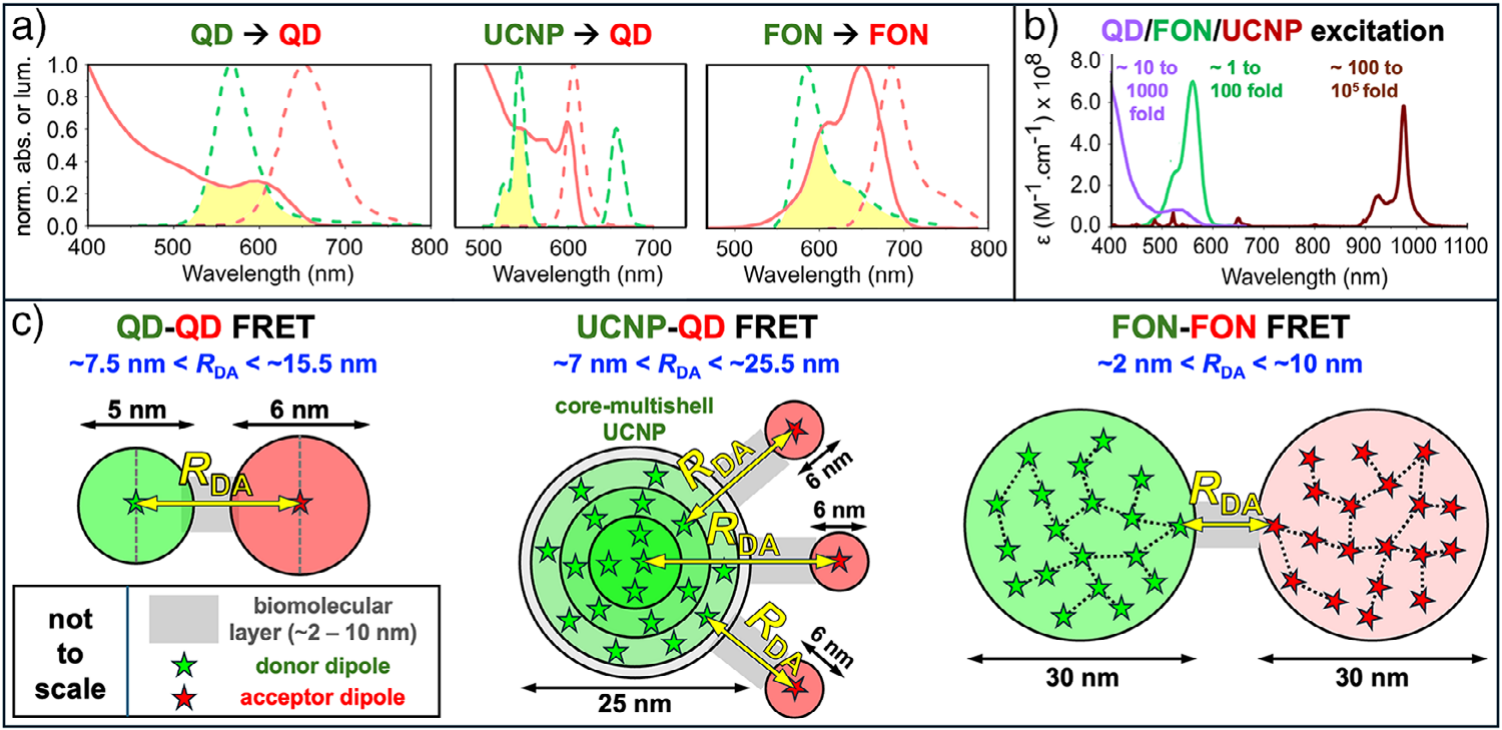
a) Intensity‐normalized absorption (abs., full lines) and luminescence (lum., dashed lines) spectra of representative inter‐NP FRET pairs. Yellow areas represent spectral overlaps. b) Molar extinction coefficient (ε) spectra of typical QDs, FONs, and UCNPs. The ε values were normalized, and typical multiplication factors to obtain that normalization (“x fold”) are shown next to the spectra. Values for a) and b) were extracted from Refs. [29, 30, 31, 32] using the open access Automeris.io web tool. c) Schematic presentation of how NP sizes and donor/acceptor positions in the NPs affect R_DA_. Values of NP sizes and biomolecular layer thickness are representative examples. For QDs, donor and acceptor can be approximated as point dipoles in the QD center, such that R_DA_ is larger than the sum of the QD radii. For UCNPs, only the lanthanide ion emitters are shown. As they are distributed throughout the core and/or the shells (the gray shell represents an undoped shell), most donors do not efficiently participate in FRET. For FONs, dyes must efficiently transfer (homo‐FRET or Dexter) energy to the surface for efficient FRET.

The major inter‐NP FRET systems provide significant spectral overlap (*J*), which is an important necessary FRET requirement (Figure 1a). Luminescence quantum yields can be high (>0.5) for QDs and FONs but are usually low for UCNPs (<0.01). For UCNP donors, Φ_D_ is the quantum yield of the emitting lanthanide ion (and not the one of the entire UCNP), which is difficult to determine,^[^ ^28^ ^]^ thus leading to a significant uncertainty for calculating *R*_0_. For FON acceptors, ε_A_ may be the value of a single dye (because the actual FRET process happens between two single dyes) or that of the entire FON, thus leading to a significant uncertainty for calculating *J*. The orientation factor κ^2^ and the refractive index *n*_r_ can also be significantly different in inter‐NP FRET systems. For example, it would be interesting to investigate the influence of luminescent NPs with non‐isotropic polarization or orientation kinetics and luminescence lifetime on κ^2^. Studying the influence of the various refractive indices between the donor and acceptor dipoles on FRET would also be intriguing. However, these parameters are still highly unexplored for inter‐NP FRET.

In addition to the photophysical parameters for FRET, the actual excitation efficiency of the donor is another important property. There is a huge difference between the absorption cross sections of FONs, QDs, and UCNPs (Figure 1b). The excitation wavelength plays an important role as well. UCNPs can be excited in the NIR with the important benefit of strongly reduced autofluorescence background in biological samples and deeper tissue penetration. QDs have a very broad absorption spectrum and can therefore be excited at any wavelength, which provides important flexibility for designing the FRET system. Brightness (*B*, Equation 5) is also a key parameter. Whereas in QDs there is only one extinction coefficient and quantum yield (though it can change with excitation wavelength), UCNPs and FONs possess many absorbers and emitters within their NP volume. In these cases, the NP brightness scales with the number (*x*) of luminescent dopants per NP (Equation 6).

(5) B=εΦ

(6) $$B_{\mathrm{NP}}=x\varepsilon \Phi$$

Conventional organic dyes have a maximum brightness of ca. 10^5^ M^−1^cm^−1^ and are hardly detectable at concentrations below 50 nM using a regular spectrofluorometer.^[^ ^33^ ^]^ QDs or FONs can easily reach brightness values above 10^6^ or 10^7^ M^−1^cm^−1^ and be detected at significantly lower concentrations.^[^ ^34^, ^35^ ^]^ NP‐based biosensors can be more diluted for decreased bioreceptor (e.g., antibody, aptamer) concentration, which can lead to lower analyte detection limits.^[^ ^36^, ^37^ ^]^ NPs with the highest absorbance tend to be the largest, which may compromise the FRET efficiency. Brightness per volume (*V*) identifies the most compact and brightest NPs (Equation 7)^[^ ^38^ ^]^:

(7) $$B_{V}=B_{\mathrm{NP}}\cdot V^{-1}$$

Very bright NPs (e.g., QDs, FONs) have *B_V_* values in the 10^3^ M^−1^cm^−1^nm^−3 [^ ^24^, ^34^ ^]^ or even in the 10^4^ M^−1^cm^−1^nm^−3[^ ^39^, ^40^ ^]^ range. Being very small in size, organic dyes exhibit an even higher brightness per volume (ca. 10^5^ M^−1^cm^−1^nm^−3^). UCNPs have by far the lowest brightness because of their relatively low quantum yields and the very low absorption cross sections of lanthanide ions. A typical value for the Yb^3+^ ion is 10^−20^ cm^2^, which corresponds to an ε of around 3 M^−1^cm^−1^ and is 3 to 4 orders of magnitude lower than organic dyes.^[^ ^41^ ^]^ For an UCNP of 20 nm diameter with a unit cell size of the NaGdF_4_ host of 0.1 nm^3^, a Yb^3+^ doping concentration of 20%,^[^ ^42^ ^]^ and an estimated quantum yield of 1%, *B_V_* would be 0.1 M^−1^cm^−1^nm^−3^. This value is again many orders of magnitude lower than the value of QDs or FONs and might become even lower in biological media. However, in real‐life applications, such as biosensing and especially bioimaging, the signal‐to‐background ratio is much more important than brightness. Near infrared (NIR) excitation of UCNP‐based probes creates many orders of magnitude lower background than excitation of QDs or FONs in the UV or visible spectral range, and NIR light can also penetrate deeper into biological tissues.

### 2.2 Distance and Size

Because of the limited distance range (usually *R*_DA_ < 10 nm), the NP size strongly influences FRET (Figure 1c). Although NP sizes can also be measured via FRET,^[^ ^43^ ^]^ the commonly used methods are transmission electron microscopy (TEM) and dynamic light scattering (DLS). TEM measures the dry diameter of objects with strong electron contrast, whereas DLS measures the hydrodynamic (including surface ligands and hydration shell) diameter. Therefore, DLS usually results in larger NP sizes. When considering spheric NPs, core diameters are usually below 10 nm for QDs, 20 nm or larger for UCNPs, and 20 nm or larger for FONs. Especially for UCNPs and FONs, the size range is very broad, and 25 and 30 nm (as shown in Figure 1c) should be understood as typical sizes of such NPs used for FRET. The precise sizes of the NPs in a biological environment are difficult to obtain because colloidal NP synthesis results in a size distribution, surface ligands (usually below 1 nm for small molecules^[^ ^44^ ^]^), coatings (e.g., ∼2 to 10 nm for 0.5–2 kDa poly(ethylene glycol)^[^ ^45^ ^]^), and biomolecules (∼2 to 20 nm depending on the biomolecules^[^ ^46^ ^]^) further increase size and distribution. Even if the NP diameter is known, determination of *R*_DA_ is not straightforward because luminescent NPs may consist of a single transition dipole that can be approximated as a point in the center of the NP (which is valid for not too large QDs)^[^ ^19^ ^]^ or of many transition dipoles distributed all over the NP (which is the case for dye‐doped NPs and UCNPs). Thus, the radii of QDs are always contributing to *R*_DA_, and many donors (in UCNPs and FONs) and acceptors (in FONs) are beyond the FRET distance range and cannot contribute to FRET. Energy migration (usually FRET or Dexter exchange between lanthanide ions in UCNP or dyes in FONs) to the surface can lead to small *R*_DA_ values. Achieving efficient energy migration requires careful optimization of lanthanide or dye doping to avoid self‐quenching within the NP. In FONs, such processes are usually very efficient due to the dyes’ close proximity and small Stokes shift, which support efficient FRET and Dexter exchanges. In opposition, UCNPs often show less effective energy migration to the surface as the different lanthanide ions (so‐called sensitizers and activators) are doped within the same UCNP. In both FONs and UCNPs, donors and acceptors close to the surface may not be ideal for FRET because they are also strongly affected by solvent quenching. Overall, the size, shape, architecture (core/shell structures), and doping scenarios of donor and acceptor NPs make FRET quite complicated, and *R*_DA_ distances beyond the FRET range are highly probable.

## 3 FRET with Quantum Dots

QDs are crystalline NPs of ca. 2 to 10 nm diameter (QDs are often assumed to be spherical) made of semiconductor materials.^[^ ^18^, ^19^ ^]^ For a given material composition, smaller QDs absorb and emit at shorter wavelengths (higher energies), and sufficiently small and spherical QDs can be approximated as point dipole emitters with isotropic emission. QDs have relatively narrow and symmetric emission bands and a very broad absorption that starts just below the emission peak and continuously grows toward shorter wavelengths (Figure 1b), reaching high molar absorption coefficients (ca. 10^5^–10^7^ M^−1^cm^−1^). QDs have been used as FRET donors and acceptors, including multiplexed and multistep FRET, and QD‐based FRET biosensing and bioimaging have been extensively reviewed in the recent literature.^[^ ^19^, ^20^, ^47^, ^48^, ^49^ ^]^ Important aspects to keep in mind are that the broad and very strong absorption and the tunable emission of QDs allow them to be paired with i) many different acceptors for good spectral overlap and minimal direct acceptor excitation and ii) long‐PL‐lifetime (e.g., lanthanides) or bioluminescent donors (to circumvent the direct QD excitation) for very large *R*_0_ values and decreased sample autofluorescence.

QD synthesis is typically done at high temperatures in nonpolar solvents, using hot injection of precursors, resulting in nanocrystals coated with nonpolar ligands.^[^ ^50^, ^51^ ^]^ For higher brightness, QDs are often core/shell semiconductor NPs. The outer shell, commonly made of ZnS (typically of 2–7 monolayers,^[^ ^52^ ^]^ with ca. 0.3 nm for each ZnS monolayer^[^ ^51^, ^53^ ^]^), facilitates the exchange of hydrophobic for hydrophilic ligands (e.g., multi‐thiols or imidazoles), allowing QDs to be used in aqueous media for biosensing.^[^ ^52^ ^]^ Bioreceptors can be attached to QDs, e.g., chemically through surface ligands or via polyhistidine‐mediated self‐assembly with the Zn‐rich QD surface.^[^ ^54^, ^55^ ^]^ The multilayered QD structure can lead to large *R*_DA_ and thereby limit *E*_FRET_.

## 4 FRET with Lanthanide Nanoparticles

Nanomaterials doped with lanthanide (Ln) ions offer a different way to obtain luminescence in the UV–vis‐NIR range.^[^ ^56^ ^]^ The unique optical properties of trivalent Ln ions allow for a variety of applications,^[^ ^57^ ^]^ including biosensing.^[^ ^58^ ^]^ Since electronic transitions are highly unfavorable for Ln, they have very low molar absorption coefficients (generally < 10 M^−1^ cm^−1^)^[^ ^59^ ^]^ but exceptionally long excited‐state lifetimes (typically in the µs to ms range), according to the Strickler–Berg law.^[^ ^60^ ^]^ The most common techniques for the synthesis of Ln‐doped NPs are hydrothermal, co‐precipitation, and thermal decomposition synthesis methods.^[^ ^61^ ^]^ Most of these methods produce particles with hydrophobic ligands, making them non‐dispersible in water. Ligand exchange or growth of a silica shell are two examples of rendering Ln NPs compatible with FRET biosensing.^[^ ^61^ ^]^ Ln‐doped NPs can provide Stokes (i.e., downshifting) and anti‐Stokes (i.e., upconversion) luminescence, both of which have been used in FRET systems. Quantum cutting (i.e., downconversion) is also possible but less relevant for FRET biosensing applications. In the case of downshifting, the most common scenario is the use of the Tb^3+^‐doped NPs as FRET donors, since Tb^3+^ emits a series of bands in the visible region upon UV excitation and can therefore be combined with different FRET acceptors.^[^ ^27^ ^]^ Representative examples are summarized in Table 1.

**Table 1:** Representative examples of FRET with downshifting Ln NPs.

Donor	Acceptor	*R*_0_ (nm)	*E*_FRET_ (%)	Analyte	Detection range	LOD	Refs.
LiYF_4_: Tb^3+^, Ce^3+^	fluorescein sodium	nd	nd	Glyphosate, ATP	0–100 µM	0.78 µM	[62]
CaF_2_: Tb^3+^, Ce^3+^	FITC	nd	nd	Avidin, suPAR	Avidin: 0.1–430 nM suPAR: 0.5–800 nM	Avidin: 164 pM; suPAR: 328 pM	[63]
KGdF₄: Tb^3+^	FITC	nd	nd	Avidin	4.5–600 nM	5.5 nM	[64]
NaYF_4_: Tb^3+^, Ce^3+^	FITC	nd	nd	Avidin	5–400 nM	4.8 nM	[65]
LaF₃: Tb^3+^	ATTO 610	5.4 ± 0.3	nd	Biotin	nd	nd	[66]
NaYF_4_: Tb^3+^, Ce^3+^	FAM	5.4	nd	Salmonella typhimurium	100–10⁶ CFU·mL⁻¹	25 CFU·mL⁻¹	[67]
LaPO₄: Ce^3+^, Tb^3+^	AuNPs (quenchers)	nd	nd	Model system	nd	nd	[68]
LaF₃: Tb^3+^	Rose Bengal	nd	85	^1^O₂ generation	nd	nd	[69]
NaYF_4_: Tb^3+^	Rose Bengal	nd	99.7	^1^O₂ generation	nd	nd	[70]
poly(PTEu)‐PAA	Ligand displacement	nd	nd	DPA	0–1.66 mM	0.30 µM	[71]
YVO₄: Eu^3^⁺, LaPO₄: Ce^3^⁺, Tb^3^⁺	AuNPs	nd	95 (YVO₄:Eu), 90 (LaPO₄:Ce,Tb)	Model system	nd	nd	[72]
YVO₄: Eu^3^⁺	Cy5	4.19	>80	Model system	nd	nd	[73]
GdVO₄: Eu^3^⁺	Methylene Blue	3.65	90	^1^O₂ generation	nd	nd	[74]

nd: not determined.

UCNPs have arguably become the most widely used type of Ln‐based nanomaterial for FRET. Because UCNPs are usually excited in the NIR (typically around 980 or 808 nm, Figure 1b), there is no direct excitation of the acceptors, and low background signals can be accomplished without the use of time‐resolved (or time‐gated) luminescence detection. Compared to UV or visible, NIR radiation also causes less photodamage to tissues and experiences lower scattering and absorption by biological media, allowing for deeper optical penetration in such biological materials.^[^ ^75^ ^]^ Although UCNPs have many properties that make them attractive for FRET biosensing applications, there are still challenges to overcome. Because UC is a non‐linear process, its efficiency depends on the excitation intensity.^[^ ^76^ ^]^ Therefore, high UC efficiencies can only be obtained at relatively high excitation power densities (>10 W cm^−2^). Such strong light excitation is not ideal for biological systems because water can be efficiently heated by NIR radiation.^[^ ^77^ ^]^ While there are several ways to obtain UC emission, the most common practice is the use of sensitizer/activator ion pairs, such as Yb^3+^/Er^3+^, Yb^3+^/Tm^3+^, or Yb^3+^/Ho^3+^. The sensitizer‐to‐activator energy transfer UC mechanism is relatively efficient (compared to other UC mechanisms) and usually requires a high ratio of sensitizers‐to‐activators.^[^ ^78^ ^]^ One of the highest reported luminescence quantum yields for such types of UCNPs was reported to be 11% and obtained in β‐NaYF_4_ NPs doped with 21.4 mol% of Yb^3+^ sensitizers and 2.2 mol% Er^3+^ activators at a pump intensity of 35 W cm^−2^.^[^ ^79^ ^]^ The addition of a protective (undoped) shell is crucial for obtaining bright UC emission. However, such shells are counterproductive for FRET because the Ln ion donors in the core part of the NP are placed at a longer distance from possible acceptors on the UCNP surface. Thus, UCNP shell thicknesses need to be carefully considered for FRET applications and should be as thin as possible while still providing adequate protection of the core‐UCNP from solvent‐quenching.^[^ ^80^, ^81^, ^82^, ^83^, ^84^, ^85^ ^]^ For example, we showed that solvent‐related luminescence quenching of UCNPs could penetrate ca. 4 nm below the surface but that a compromise between optimal FRET and adequate solvent protection could be best accomplished with protective shells in the 1.5 to 3 nm thickness range.^[^ ^83^, ^86^ ^]^ While these values may change with the size, composition, and architecture of the UCNP, they provide a good guideline for designing optimal shell thicknesses for FRET.

In addition to FRET with dye acceptors, UCNPs have also been used as NSET donors to AuNP acceptors. As AuNPs are not luminescent (their strong plasmon absorption band in the 450–650 nm wavelength range is used for luminescence quenching),^[^ ^87^ ^]^ we only summarize some examples of such UCNP‐to‐AuNP NSET biosensing systems here. The combination of UCNPs with AuNPs has been used to quantify pathogenic bacteria,^[^ ^88^ ^]^ SARS‐CoV‐2 RNA,^[^ ^89^ ^]^ detect Hg^2+[^ ^90^ ^]^ as well as many other applications.^[^ ^91^, ^92^, ^93^ ^]^ It is important to remember that the NSET acceptor is the surface (because of the localized surface plasmon resonance) of an AuNP, which means that the distance between the Ln ion donors and the AuNP surface is independent of the AuNP size.

## 5 FRET with Fluorescent Organic Nanoparticles

Similar to UCNPs, FONs contain multiple fluorophores. Energy transfer phenomena between chromophores of the same type (such as homo‐FRET or homo‐Dexter transfer) occur within the NP, making the spectral signature more complex than for single emitters like QDs. FRET between two different dyes encapsulated within the same organic NP^[^ ^94^, ^95^, ^96^, ^97^, ^98^ ^]^ or in a core/shell structure^[^ ^99^ ^]^ are not covered here and we only describe FONs architectures that have been used for FRET between NPs. Considering their high *B_V_* values,^[^ ^34^, ^100^ ^]^ FONs can potentially be excellent FRET donors or acceptors. The high packing density of fluorophores in FONs involves very efficient energy migration. For polymers, this energy migration can occur within a single chain or between polymer chains. Typical exciton diffusion lengths in dense amorphous organic samples are between 10–70 nm.^[^ ^101^, ^102^ ^]^ Thus, for sufficiently small NPs, a single exciton can travel the entire NP before emitting light or finding a FRET acceptor. For biosensing applications, this implies that excitons generated by photoexcitation can efficiently migrate^[^ ^103^ ^]^ to the “FRET position” (proximity to an acceptor) of the NP.^[^ ^95^, ^99^, ^104^, ^105^ ^]^ Funneling the excitation energy of many fluorophores via energy migration to the FRET donor and then via energy transfer to the FRET acceptor can significantly amplify the acceptor fluorescence and thereby increase biosensing sensitivity.^[^ ^106^, ^107^ ^]^

Pdots are a sub‐category of conjugated polymer NPs that are made of hydrophobic semi‐conducting fluorescent polymers (weight‐% >50%, ideally > 80%^[^ ^18^ ^]^) with diameters ideally below 30 nm but reported up to 100 nm.^[^ ^23^, ^108^, ^109^, ^110^ ^]^ The polymers are often composed of alternating electron donor and acceptor monomers (e.g., fluorene, benzothiadiazole) linked by π‐bridges (e.g., thiophene, phenyl, ethylene), which impart large Stokes shifts (2800–3800 cm^−1^). Pdots usually have broad absorption bands in the 250 to 500 nm wavelength range and emit between ca. 430 and 600 nm with broad bands (FWHM ∼40–200 nm). The absorbance of Pdots increases with their size, and *B_V_* values are in the order of 10^3^–10^4^ M^−1^cm^−1^nm^−3^. Luminescence quantum yields can vary between 5% and 60%, and luminescence lifetimes are short (τ < ns). The conjugated polymeric backbones are hydrophobic, and therefore aggregates of polymers cannot be used for biosensing. A common strategy to obtain NPs from the conjugated polymers is to co‐nanoprecipitate them with an amphiphilic copolymer, such as polystyrene‐*b*‐poly(ethylene glycol) or poly(ethylene glycol)‐phospholipid derivatives. Bioconjugation can be performed through classical chemical reactions (e.g., click chemistry, peptide coupling) using accessible appendices at the amphiphilic polymer extremities.

Pdots have only been used as FRET donors, which is likely related to their absorption at relatively short wavelengths (250 to 500 nm). It has been demonstrated that energy transfer to Au‐NPs (NSET),^[^ ^111^ ^]^ QDs,^[^ ^112^ ^]^ or carbon nanotubes^[^ ^113^ ^]^ is possible. However, when it comes to biosensing applications, Pdots are mainly associated with organic dyes.^[^ ^40^, ^98^, ^114^, ^115^ ^]^ Lix et al. highlighted the importance of the position of a molecular organic acceptor (Cyanine5 or Cyanine7, ε_A_∼2 × 10^5^ M^−1^cm^−1^) to maximize FRET efficiency.^[^ ^40^ ^]^ While very efficient FRET occurs from a single Pdot to multiple acceptors when the dye is immobilized directly on the Pdot (*E*_FRET_ ∼10%–30%) it drops to less than 5%–10% when the acceptor is immobilized on a bioreceptor. Pdots usually have an amphiphilic polymer corona for NP stabilization (∼1.5 nm) and an oxidized semi‐conducting polymer corona (0 to 10 nm). Both are localized between the fluorescent core and the FRET acceptors on the surface, which is disadvantageous for FRET.^[^ ^116^ ^]^ As energy migration is not permitted in those coronas, it limits energy transfer to the acceptor on the bioreceptor. Fluorescent conjugated polymers can be rendered amphiphilic by the introduction of charges on certain monomers and thus can self‐assemble in water into micelle‐like structures.^[^ ^117^, ^118^ ^]^ It was demonstrated that such structures can be used as NSET donors to Au‐NP acceptors^[^ ^119^, ^120^ ^]^ or FRET donors to charged molecular dyes, such as ethidium bromide.^[^ ^121^ ^]^

PNPs^[^ ^24^, ^100^, ^122^ ^]^ are typically synthesized via nanoprecipitation of amphiphilic copolymers^[^ ^123^ ^]^ or by polymerization in dispersed media^[^ ^124^ ^]^ whether the dye is trapped in the hydrophobic polymer matrix^[^ ^125^, ^126^ ^]^ or copolymerized.^[^ ^127^, ^128^ ^]^ Common dyes found in such structures include BODIPY,^[^ ^128^ ^]^ rhodamine,^[^ ^126^ ^]^ fluorescein,^[^ ^129^ ^]^ and cyanine^[^ ^129^ ^]^ derivatives, which exhibit high molar absorptivity and quantum yield even in the solid state but have a small Stokes shift (e.g., 500–1000 cm^−1^). Larger molecules with a more extended π‐system, typically of a push–pull nature,^[^ ^130^, ^131^, ^132^ ^]^ can also be incorporated, offering the advantage of a larger Stokes shift (e.g., 5000–7000 cm^−1^) for easier selective donor excitation, with the drawback of usually less efficient energy migration within the NPs. In PNPs, the dyes are trapped in a hydrophobic polymer matrix, preventing their aggregation and rotation and reducing non‐radiative deactivation. The fluorescence quantum yield, lifetime, and Stokes shift directly depend on the nature of the encapsulated dye. Quantum yield can vary from a few percent to up to 80%^[^ ^34^, ^100^ ^]^ and lifetimes rarely exceed 5 ns, though recent combinations of dyes provided lifetimes above 20 ns.^[^ ^94^ ^]^ To disperse PNPs in water, it is required to cover them with hydrophilic or amphiphilic polymers, which also serve for bioconjugation. Similar to Pdots, PNPs are most often used as FRET donors for organic dyes^[^ ^104^, ^133^ ^]^ or AuNPs (NSET).^[^ ^134^ ^]^ Some examples report the use of PNPs as FRET acceptors in combination with organic dyes.^[^ ^135^ ^]^ Klymchenko et al. showed that PNPs doped with bulky rhodamines are very efficient light harvesters.^[^ ^95^, ^104^ ^]^ On average, 3200 rhodamine donors were embedded in 40 nm PNPs, and with a Ф_D_ of 47%, the maximum *B_V_* was estimated around 5.6 × 10^3^ M^−1^cm^−1^nm^−3^. The high density of dyes in the NPs allowed for efficient energy collection and migration to a small number of acceptors (Cyanine5). The brightness of a single dye acceptor inside the donor NPs could be amplified 1000‐fold, and when the dye was attached to the PNP surface via short oligonucleotides, ratiometric DNA quantification resulted in a limit of detection of 5 pM. Drastic fluorescence quenching of an entire PNP (BODIPY‐loaded) by organic dyes (methylene blue) was also shown by Si et al. to distinguish between NPs localized inside or outside bacteria.^[^ ^133^ ^]^

Cdots^[^ ^25^ ^]^ are carbon‐rich NPs smaller than 10 nm in diameter that can be synthesized using top–down or bottom‐up approaches. The origin of their luminescence remains a subject of debate, and optical properties are varied using different percentages of oxygen or nitrogen as dopants and carbon crystallinity in the core. Cdots exhibit strong absorption in the UV, which is attributed to π–π* and n−π* transitions associated with alkene, amide, and ester functions, more or less conjugated. One distinctive feature of Cdots is their excitation wavelength‐dependent luminescence, caused by the diversity of luminescent moieties in their core. This characteristic can be exploited to enhance FRET but can also significantly complicate the interpretation of spectroscopic data. Cdots are highly water‐soluble, often due to their surface carboxylic acid functions that also facilitate their bioconjugation.^[^ ^136^ ^]^ Given that CDots do not require surfactants for stabilization, potential acceptors on the surface will consequently be closer compared to QDs, UCNPs, or FONs and result in possibly higher FRET efficiencies. Donor–acceptor distances can even be sufficiently short for photoinduced electron transfer. Owing to their small size, ease of synthesis, and simple surface functionalization, Cdots have been implemented in many biosensors^[^ ^137^, ^138^ ^]^ despite their relatively low brightness (usually 10 to 100 times lower than QDs or FONs of comparable size).^[^ ^18^ ^]^ Due to their excitation at ∼400 nm and blue emission, Cdots have primarily been used as energy donors,^[^ ^139^ ^]^ mainly combined with either Au‐NPs (NSET)^[^ ^140^, ^141^, ^142^, ^143^ ^]^ or organic dyes.^[^ ^144^, ^145^ ^]^ While being a relatively new field, it has already been demonstrated that FRET with Cdots can be used to quantify a number of relevant analytes, including metals ions,^[^ ^146^, ^147^ ^]^ biological thiols,^[^ ^140^, ^148^ ^]^ nucleosides,^[^ ^142^ ^]^ glucose,^[^ ^145^ ^]^ and enzymes.^[^ ^144^ ^]^

## 6 Inter‐Nanoparticle FRET

Although it can be expected that the constant development of ever more luminescent nanomaterials will also lead to more inter‐NP FRET, it is currently a relatively small and specific sub‐field of FRET. The 25 examples summarized in Table 2 provide a representative overview of inter‐NP FRET systems developed for sensing in water. Two main approaches to biosensing can be distinguished: i) Approximately a quarter of the studies^[^ ^149^, ^150^, ^151^, ^152^, ^153^ ^]^ employ a strategy in which the analyte affects the emission of one of the fluorophores in the assay (typically the acceptor) without altering the donor–acceptor interaction; ii) the majority of analytical nanosystems detect the analyte through changes in the actual FRET rate. While ca. 90% of the articles reported the NP diameters, only 40% and 60% provided *R*_0_ and *E*_FRET_ values, respectively, and only ca. 70% specified the biosensor's limit of detection. The absence of such figures of merit makes it difficult to directly compare the different systems. The following sections provide a more detailed and critical analysis of each FON/FON, QD/QD, UCNP/QD, and other NP/NP FRET donor/acceptor pair. All examples show that the competition of photophysics versus size is highly relevant but that appropriate knowledge of the limitations, sophisticated design, and ingenious exploitation of NP benefits can result in unique FRET systems that are not affordable with other materials. The FRET‐relevant properties of QDs, UCNPs, and FONs are summarized in Table 3.

**Table 2:** Overview and key properties of inter‐NP FRET pairs for biosensing.

D/A^a)^	D/A dia‐meters nm	*R*_0_ nm	*E*_FRET_ %	Analyte	Detection range	LOD^b)^	Year	Refs.
QD/QD	2.5/5.0	nd	40^c)^	BSA	Nd	10^−8^ M	2002	[154]
QD/QD	∼2/3	6.5	41	Mouse IgG	1 × 10^−4^–2 × 10^−2^ g L^−1^	4.2 × 10^−5 ^g L^−1^	2005	[155]
QD/QD	4.1/6.4	5.4	50^c)^	K^+^	Nd	10^−6^ M	2006	[156]
QD/QD	nd	6.6	51	Rabbit IgG	Nd	nd	2007	[157]
QD/QD/QD	2.5/3.2/5.5	6.3/7.6/8.2	>90^c)^	Ca^2+^	Nd	3.3 × 10^−5^ M	2008	[158]
QD/QD	2.2/5.3	nd	45	CD71 antigen ^d)^	Nd	nd	2008	[159]
QD/QD	4/4 × 9	nd	25^c)^	TNT	5 × 10^−12^ −5 × 10^−7^ M	5 × 10^−12^ M	2010	[160]
QD/QD	nd	nd	58	protease	Nd	nd	2011	[161]
QD/QD	nd	nd	50	*Salmonella*	5–75 × 10^5^ CFU mL^−1^	10 CFU mL^−1^	2014	[162]
QD/QD	2.3/3.5	nd	75^c)^	DNT	1–7 × 10^−4^ M	nd	2019	[163]
QD/QD	2/2	nd	50^c)^	miRNA	20–100 × 10^−12^ M	14 × 10^−12^ M	2020	[164]
QD/QD	2.3/1.7	5.4	66	H^+^	−	−	2021	[30]
UCNP/QD	16/2.6	nd	21	Pb^2+^	0.02–3.6 × 10^−6^ M	8 × 10^−8^ M	2014	[151]
UCNP/QD	17.1/3.5	nd	28	Hg^2+^	0.01–2.8 × 10^−6^ M	15 × 10^−9^ M	2015	[152]
UCNP/QD	24.2/∼10	nd	nd	HPRT1	5 × 10^−9^–2 × 10^−6^ M	13 × 10^−15^ M	2015	[165]
UCNP/QD	∼30/16	6.0	7	Biotin	Nd	5 × 10^−9^ M	2015	[31]
UCNP/QD	100/∼3	3.2	33	procalcitonin	0.01–1 × 10^−5^ g L^−1^	2.5 × 10^−7^ g L^−1^	2018	[166]
UCNP/QD	25/3.5	nd	>90^c)^	MPP2	10^−3^–1 × 10^−11^ g L^−1^	nd	2018	[167]
Pdot/Pdot	94/94	6.0	nd	ssDNA	1 × 10^−14^–1 × 10^−12^ M	∼10^−14^ M	2015	[168]
PNP/PNP	40/25	6.4	97	ssDNA	1–100 × 10^−12^ M	0.36 × 10^−12^ M	2023	[32]
Cdot/Cdot	∼7/∼7	nd	61	Oxygen	Nd	nd	2017	[153]
gQD/Cdot	3.3/3.8	5.1	39	As^5+^	30–200 × 10^−6^ M	nd	2019	[150]
Cdot/Cdot	6.5/5.1	nd	nd	PCT	0.5–5 × 10^−6^ g L^−1^	0.3 × 10^−6^ g L^−1^	2025	[169]
gQD/QD	3/5	∼5^c)^	57	chlortoluron	2.4 × 10^−10^–8.5 × 10^−8^ M	7.8 × 10^−11^ M	2015	[149]
gQD/QD	10/5	nd	41	Insulin	3.2 × 10^−8^–2.5 × 10^−4^ g L^−1^	4.5 × 10^−8^ g L^−1^	2022	[170]

^a)^ Donor/acceptor pair.

^b)^ Limit of detection.

^c)^ Data estimated by the authors based on maximum donor fluorescence quenching (steady‐state) upon acceptor addition.

^d)^ Cell membrane labelling.

gQD: graphene QD.

**Table 3:** FRET‐relevant properties of QDs, UCNPs, and FONs.

	QDs	UCPN	FONs
***D* (nm)**	2–10 [18]	10–100 [18]	2–100 [18]
**ε (M^−1^ cm^−1^)**	10^5^–10^7^ [18]	10^4^–10^7^ [18]	10^6^–10^9^ [18]
**Φ_F_ (%)**	10–80 [18]	≤10 [18]	5–60 [18]
***B_v_* (M^−1^ cm^−1^ nm^−3^)**	10^3^	∼0.1	10^3^–10^4^
**Δυ (cm^−1^)** ^a)^	5000–12 000	5000–8000 [76]	800–6000 [24]
**τ (s)**	10^−9^–10^−6^ [18]	10^−4^–10^−2^ [18]	10^−10^–10^−9^ [18]
**Photostability**	Excellent	Excellent	Moderate
**Used as donor?**	Yes	Yes	Yes
**Used as acceptor?**	Yes	No	Yes

^a)^ (Anti‐)Stokes shift: for the QDs, Stokes shift is estimated using an excitation wavelength of 405 nm, for the UCNPs, anti‐Stokes shift is estimated using an excitation wavelength of 980 nm and emission of Er^3+^.

### 6.1 FRET Between QDs

While QDs have been widely used as both energy donors (QD_D_) and acceptors (QD_A_),^[^ ^171^, ^172^, ^173^, ^174^ ^]^ their combination within one FRET pair is more challenging.^[^ ^175^ ^]^ Because QDs absorb all wavelengths below their emission wavelength and the donor emission must overlap with the acceptor absorption, both QD_D_ and QD_A_ are always simultaneously excited by the excitation light (Figure 1a,b). For QDs of the same material, the redder QD_A_ will absorb even more light than QD_D_.^[^ ^176^ ^]^ Thus, the excited‐state donor and ground‐state acceptor requirement for FRET is not fulfilled. It does not mean that QD_D_‐to‐QD_A_ FRET is impossible, but it is difficult to measure. The first studies describing FRET between aggregated CdSe QDs in films date back to 1996.^[^ ^177^ ^]^ These studies were followed by a series of investigations on various types of QDs (e.g., silicon QDs,^[^ ^178^ ^]^ CdTe,^[^ ^179^, ^180^ ^]^ CdSe/ZnS,^[^ ^181^ ^]^ CdSe/ZnCdS,^[^ ^181^ ^]^ CdSe,^[^ ^177^, ^182^ ^]^ ZnSe,^[^ ^180^ ^]^ CdSe/ZnS,^[^ ^183^ ^]^ CdSe/ZnTe^[^ ^183^ ^]^) demonstrating both homo‐ and hetero‐transfer in solid films or in aerogel‐type matrices.^[^ ^180^ ^]^ They highlight that FRET between QDs of the same or different types is indeed possible and always occurs from larger to smaller bandgap QDs (most often smaller to larger QDs). Erdem et al.^[^ ^184^ ^]^ demonstrated that FRET between spherical QDs (radial transition dipole) and nanoplatelet surfaces (axial transition dipole) follows an *R*_DA_^−4^ (NSET) rather than an *R*_DA_^−6^ (FRET) distance dependence. Similar to NSET with AuNP acceptors, the *R*_DA_^−4^ distance dependence was most likely caused by the point‐dipole to surface‐dipoles energy transfer.^[^ ^22^ ^]^ It would be very interesting to investigate if nanoplatelets would result in a similar distance extension for other NP donors. The authors also showed that the dipole orientation of the nanoplatelets modulated the orientation factor κ^2^ from 1/3 to 5/6, which also influenced the energy transfer efficiency. Such multi‐component materials hold promise for applications in white‐light‐emitting devices, displays, and more.

For biosensor applications, it is crucial to analyze a colloidal dispersion of QDs, rather than films. QD aggregates in organic solvents have been used to demonstrate the presence of FRET and presented key advancements in biosensor development. ^[^ ^178^, ^179^, ^181^, ^185^, ^186^ ^]^ QD aggregates in water, mainly involving CdSe^[^ ^187^ ^]^ or CdTe^[^ ^158^, ^176^, ^188^, ^189^, ^190^ ^]^ QDs, were also investigated, and Osovsky et al. demonstrated that energy transfer between CdTe QDs dispersed in water is experimentally feasible.^[^ ^188^ ^]^ They used QDs emitting at 575 nm (diameter 3.2 nm, Φ = 15%) and 615 nm (diameter 3.4 nm, Φ = 3%) coated with cysteamine or thioglycolic acid. *R*_0_ values were found to range between 5.5 and 10.9 nm, depending on the QD combinations. QDs were brought in proximity either by covalently linking their surface ligands (ligand layer thickness: 0.9 nm) or through electrostatic interactions (ligand layer thickness: 1.1 nm). As a result, the core‐to‐core distance was significantly shorter than *R*_0_. Under optimal conditions, FRET rates as high as 10^10^ to 10^11^ s^−1^ were observed, significantly faster than the fluorescence lifetime of the donor alone (5.5 ns). However, the long‐term water stability of such QDs remained an open question. FRET cascades between small aggregated CdTe QDs in water were also shown through electrostatic interaction with divalent cations.^[^ ^158^ ^]^ By adding [Ca^2+^] = 5 × 10^−4^ M to a mixture of QDs emitting at three different wavelengths (550, 600, and 730 nm), respective quenching rates of 96% and 22% for the green and red QDs resulted in a 40% fluorescence intensity increase of the NIR QD.

More recently, Dreesen and Humbert's teams identified key factors to optimize FRET between QDs in water,^[^ ^176^, ^189^ ^]^ including QD proximity, density of electronic states, and energy dissipation into the surrounding environment. They reasonably hypothesized that FRET would generally be more efficient with smaller QDs (e.g., CdTe) compared to larger core‐shell QDs. Nevertheless, FRET between core‐shell QDs has been observed, e.g., between positively charged ZnCdSe/ZnS QDs (λ_em_ = 518 nm, diameter 6 nm) and negatively charged CdSe/ZnS QDs (λ_em_ = 563 nm, diameter 4 nm) for which an *R*_0_ of 7.3 nm was reported. 93% quenching of the donor was observed, along with a 1.5‐fold acceptor emission enhancement (using ten donors per acceptor).^[^ ^191^ ^]^ In another example, FRET between Cd‐free InP/ZnS QDs (λ_em_ = 515 nm, diameter 3.5 nm) and CInS/ZnS QDs (λ_em_ = 630 nm, diameter 3.7 nm) resulted in 60% donor luminescence lifetime quenching.^[^ ^192^ ^]^ In aggregated systems, the photophysical interactions between NPs can become complex. Photoinduced electron (or charge) transfer between NPs, particularly at distances below 1 nm, cannot be ruled out.^[^ ^193^, ^194^ ^]^ Additionally, Dexter transfer (i.e., electron exchange) is possible at such short distances. When it becomes difficult to distinguish between the different charge or energy transfer mechanisms, the more general term “energy transfer”^[^ ^195^ ^]^ is maybe the most suitable.

**Figure 2:**
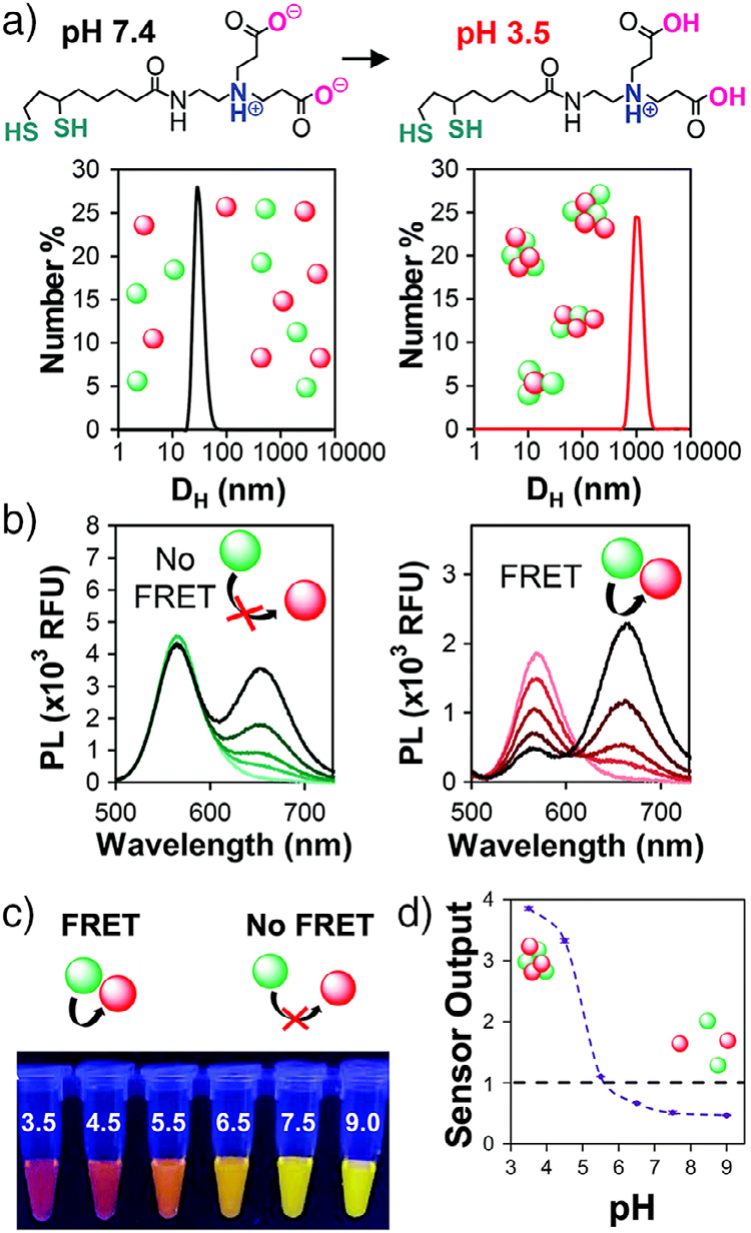
QD–QD FRET with InP/xZnSe/3ZnS donor and InP/3ZnS acceptor nanocrystals. a) donor QDs (green) and acceptor QDs (red) capped with dithiolate zwitterions. At pH 7.4, the QDs are stabilized by the carboxylates on their surface. At pH 3.5 aggregation is induced by the protonation of the carboxylic acids, as shown by increased hydrodynamic diameters. b) Fluorescence spectra of this system with acceptor‐to‐donor ratios of 0, 0.5, 1, 2, and 4, as indicated by the light‐to‐dark transition in trace color. c) Photograph of the nanosensor at different pH, showing the visible color change. d) Graphical representation of the pH‐dependent sensor output. Reproduced with permission from Ref. [30], Copyright 2020 Royal Society of Chemistry.

Many QD_D_–QD_A_ FRET sensors have made use of CdTe, CdSe/ZnS, InP/ZnS, or InP/ZnSe/ZnS QD aggregation to detect ions, such as Ca^2^⁺,^[^ ^158^ ^]^ K⁺,^[^ ^156^ ^]^ or H⁺.^[^ ^30^ ^]^ Owing to the high brightness of the QDs (110 × 10^3^ M^−1^cm^−1^ and 15 × 10^3^ M^−1^cm^−1^), QD‐FRET‐induced fluorescence color change could even be visualized with the naked eye^[^ ^30^ ^]^ (Figure 2). Other CdTe or CdSe/ZnS QD aggregates were applied for the detection of 2,4‐dinitrotoluene^[^ ^163^ ^]^ or 2,4,6‐trinitrotoluene.^[^ ^160^ ^]^ Protease detection was realized via aggregation of CdTe QDs coated with oppositely charged peptides. Protease digestion of the peptides disassembled the aggregates and led to a concomitant FRET interruption.^[^ ^161^ ^]^ The main limitation of aggregation‐based systems is their generally relatively low specificity to the target analyte, which can pose problems in complex environments such as biological samples. In addition, uncontrolled cascade FRET between the same QDs^[^ ^182^ ^]^ can make such aggregates less bright. Although homo‐FRET does not theoretically lead to energy loss, the probability of deactivation via even rare non‐fluorescent states becomes higher during a long FRET cascade.

Leveraging biomolecular recognition molecules (e.g., antibodies or DNA) can result in higher target specificity. The use of QD_D_–QD_A_ FRET‐based assays with specific biorecognition molecules was demonstrated on small CdTe QDs (∼2–5 nm) emitting green or red light (λ_em _= 555/610 nm^[^ ^154^ ^]^ or 535/590 nm^[^ ^155^ ^]^ or 573/638 nm^[^ ^157^ ^]^). These QDs were bioconjugated with either i) bovine serum albumin (BSA) and an anti‐BSA immunoglobulin G (IgG) antibody to form a QD_D_‐BSA‐IgG‐QD_A_ FRET sandwich complex,^[^ ^154^ ^]^ ii) positively charged mouse IgG to bridge two negatively charged QDs for FRET to form a QD_D_‐IgG‐QD_A_ FRET complex,^[^ ^155^ ^]^ or iii) rabbit IgG (rIgG) and goat anti‐rabbit IgG (garIgG) to form a QD_D_‐rIgG‐garIgG‐QD_A_ FRET sandwich complex.^[^ ^157^ ^]^ In those three scenarios, the FRET efficiencies were between 40% and 50% and two of the studies mentioned *R*_0_ values of ∼6.5 to 6.6 nm.^[^ ^155^, ^157^ ^]^ The QD_D_–QD_A_ FRET systems had 2:1,^[^ ^157^ ^]^ 1:4,^[^ ^155^ ^]^ and 1:1^[^ ^154^ ^]^ donor:acceptor ratios, and the authors reported the quantification of 10 nM BSA using 5 nM QD‐bioconjugates,^[^ ^154^ ^]^ of sub‐0.1 mg L^−1^ IgG using QDs at µM concentrations,^[^ ^155^ ^]^ and of IgG in the mg L^−1^ concentration range using QDs at µM concentrations.^[^ ^157^ ^]^ These studies pioneered the demonstration of QD_D_–QD_A_ FRET biosensing. However, the QD structures used, along with their molecular ligands, such as mercaptopropionic acid, did unfortunately not allow for long‐term studies (more than hours) or experiments under conditions with pH below 6.5 or varying saline concentrations.^[^ ^155^, ^157^ ^]^

Another example of CdTe QD‐based QD_D_–QD_A_ FRET with specific biorecognition molecules used a competitive immunoassay for the quantification of *Salmonella Enteritidis*.^[^ ^162^ ^]^ Green (λ_em_ = 514 nm) and orange QDs (λ_em_ = 578 nm) were bioconjugated with rabbit anti‐*S*. *Enteritidis* antibodies and goat anti‐rabbit IgG, respectively, such that they could form a QD_D_‐antibody‐IgG‐QD_A_ sandwich complex, which showed fluorescence of both QDs upon excitation at 330 nm. The energy transfer mechanism was not investigated, but because the addition of *S*. *Enteritidis* resulted in competitive binding to the anti‐*S*. *Enteritidis* antibody and an increase/decrease in the QD_D_/QD_A_ fluorescence intensities, it was assumed that FRET was present and the fluorescence intensities provided an *E*_FRET_ value of ∼50%. *S*. *Enteritidis* could be quantified in solution in a concentration range of 75 to 5 × 10^5^ colony‐forming units per mL (CFU mL^−1^) and qualitatively detected in egg samples. More stable CdSe/ZnS core/shell QDs were shown to be applicable for cellular immunostaining and imaging.^[^ ^159^ ^]^ Green (λ_em_ = 544 nm) and red (λ_em_ = 614 nm) QDs were bioconjugated with goat anti‐mouse IgGs and mouse anti‐CD71 antibodies, respectively. The QD_A_ immunoconjugate bound to CD71 on HeLa cells, and the QD_D_ immunoconjugate bound to the QD_A_ immunoconjugate, such that a color change could be observed on the cell membrane. Although the FRET mechanism was not characterized, fluorescence spectra and intensities were analyzed and showed an *E*_FRET_ of ∼45%.

More recently, a combination of specific biological recognition, i.e., DNA–RNA hybridization, and aggregation of mercaptoacetic acid‐capped CdTe QDs was used for the quantification of miRNA.^[^ ^164^ ^]^ This study efficiently demonstrated how confusing the characterization and interpretation of fluorescence from a dual‐QD system can be. The green QDs were assumed to aggregate only in the presence of the DNA–RNA heteroduplex and not when only ssDNA was present, which the authors attributed to the ability of QDs to strongly interact with double‐stranded nucleic acids but without any specific explanation why QDs would do that or any characterization of aggregates in solution. The authors further stated that electron transfer between the green QDs in the aggregate led to fluorescence quenching and that the addition of orange QDs and more miRNA resulted in FRET to further quench the green QDs while increasing the orange QD fluorescence intensity. Despite the unclear explanation of the photophysical changes and energy transfer mechanisms, the fluorescence intensity ratio resulted in an miRNA concentration‐dependent linear response in the 20 to 90 pM concentration range with a reported detection limit of 14 pM miR‐155.

QD–QD FRET has remained an academic topic of interest, most likely due to the unavoidable and very efficient light excitation of both QD_D_ and QD_A_, which disfavors FRET and makes it difficult to analyze. The relatively large size (compared to typical FRET distances) of both QD_D_ and QD_A_ is likely to be another limiting factor. Many studies focused on proof‐of‐concept demonstration and applied QD aggregation in films and organic solvents but less often in aqueous media. Some studies were able to detect and quantify biological or chemical targets, but without significant improvement compared to existing techniques or other FRET systems. Most QD–QD FRET systems employed small CdTe core‐only QDs because of their smaller sizes compared to core‐shell QDs, despite the latter having higher quantum yields and brightnesses. To gain a better understanding of QD–QD FRET for biosensing, more profound characterization, including determination of all FRET parameters, steady‐state and time‐resolved spectroscopy, and possibly modeling of the bioconjugate sizes and donor–acceptor distances, would be important. It would also be very interesting to use QD donors with much longer fluorescence lifetimes than QD acceptors, which could be realized by manganese‐doped QDs or a FRET cascade from long luminescence lifetime lanthanides to QD_D_ to QD_A_. In the case of FRET, the long lifetime should also become visible for QD_A_. The same cascade approach could also be tested with initial bioluminescent or chemiluminescent donors, which would avoid direct light excitation of QDs.

### 6.2 FRET Between UCNPs and QDs

Despite the relatively low luminescence quantum yields, large sizes, and the protecting shells of UCNPs and the point‐dipole approximation in the center of QDs, all of which are rather counterproductive for FRET, UCNP‐to‐QD FRET has been successfully demonstrated (cf. Table 2). Similar to QD–QD FRET, the smaller the QDs, the better the distance for FRET. A very important advantage of UCNP donors is their excitation in the NIR, which does not directly excite the QD acceptor, such that UCNP‐to‐QD FRET is significantly more straightforward to detect. The undoped UCNP shell should be as thin as possible. The influence of shell thickness (3 to 16 nm) on FRET was investigated both theoretically and experimentally for NaYF_4_:Er^3+^/Yb^3+^@SiO_2_ UCNP donors and CdSe QD acceptors.^[^ ^196^ ^]^ The authors showed that around 24 QD acceptors were able to attach to a single UCNP. The highest observed FRET efficiency between the Er^3+^ donors close to the UCNP surface and the QD acceptors was around 10% for a 3 nm SiO_2_ shell. For thinner shells, UCNP luminescence quenching would outcompete FRET, and for thicker shells, *R*_DA_ would be too large for FRET.

Early works on UCNP‐to‐QD FRET directly embedded QDs in the silica shells of UCNPs to perform cellular imaging,^[^ ^197^ ^]^ coated QDs on UCNPs for creating wavelength‐tunable UCNPs,^[^ ^198^ ^]^ or created UCNP‐QD heterostructures,^[^ ^199^ ^]^ all without specifically investigating the energy transfer mechanism. Bednarkiewicz et al. demonstrated UCNP‐to‐QD FRET in organic solvent but mentioned a potential application for protein/DNA assays or biopsy screening.^[^ ^200^ ^]^ By analyzing the UCNP luminescence lifetime, the FRET efficiency was estimated to be ca. 15%, with the highest contribution from Er^3+^ ions close to the surface of the particle, because the estimated *R*_0_ was only 1.5 nm. Although luminescence lifetime measurements are usually very well suited to determine FRET efficiencies, more recent work suggested that they are not adequate for such purposes and luminescence intensities may be better.^[^ ^80^, ^201^, ^202^, ^203^ ^]^ The reason is that FRET takes place simultaneously with energy migration within the UCNPs, such that the FRET donor energy level is populated and depopulated at the same time, and lifetime measurements cannot easily distinguish between the two processes.

Various other studies investigated the fundamental aspects of UCNP‐QD FRET. For example, Marin et al. studied the effect of UCNP donor architecture on *E*_FRET_ using LiYF_4_:Yb^3+^,Tm^3+^ UCNPs and CuInS_2_ QDs.^[^ ^204^ ^]^ It was shown that both the reduction of the UCNP size and the specific distribution of the QDs on the surface of the UCNPs can lead to a significant increase in *E*_FRET_. Zeng et al. investigated a combination of BaYF_5_:Yb,Ln (Ln = Er^3+^, Ho^3+^, Tm^3+^) UCNP donors and CsPbBr_3_ QD acceptors.^[^ ^205^ ^]^ They suggested that QDs could be sensitized by the UCNPs both through FRET (with *E*_FRET_ in the range of 15%–35%) and radiative energy transfer, which complicates the interpretation of the spectroscopic data. Ruan and Zhang used Yb,Tm‐doped NaYF_4_ and NaGdF_4_ UCNPs as donors for lead halide perovskite QD acceptors.^[^ ^206^ ^]^ Tuning of the anion composition of the QDs demonstrated that the energy transfer was primarily radiative in nature. The effect of the temperature on *E*_FRET_ was investigated in the work by Zhang et al.^[^ ^207^ ^]^ They investigated *E*_FRET_ between NaYF_4_:Yb^3+^, Er^3+^@NaYF_4_ UCNPs, and ZnCdSe/ZnS QDs within a temperature range of 77–427 K. It was shown that *E*_FRET_ was temperature‐dependent with a minimum at 317 K. This was most likely caused by temperature‐dependent variation of the Förster distance.

**Figure 3:**
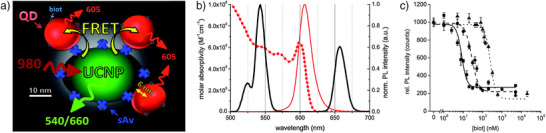
a) Schematic of UCNP‐to‐QD FRET upon UCNP‐sAv‐biot‐QD binding. UCNP excitation at 980 nm led to UCNP emission around 540 and 660 nm or UCNP‐to‐QD FRET and QD emission around 605 nm. b) Luminescence (solid lines) emission spectra of UCNP‐sAv (black) and biot‐QD (red) and molar absorptivity spectrum of biot‐QD (red dotted line) showing the spectral overlap of UCNP emission and QD absorption as well as the distinct spectral emission ranges of UCNP and QD. c) Calibration curves for a competitive homogeneous assay for biotin. 75 (solid line), 300 (dashed line), and 1200 pM (dotted line) UCNP‐sAv in the presence of 250, 1000, and 4000 pM of biot‐QD, respectively. Reproduced with permission from Ref. [31], Copyright 2015 Royal Society of Chemistry.

In 2015, several studies described actual biosensing applications using UCNP‐to‐QD FRET.^[^ ^31^, ^152^, ^165^ ^]^ One prototypical assay quantified biotin in a competitive FRET assay using streptavidin (sAv)–coated NaYF_4_:Yb^3+^,Er^3+^ UCNPs as donors (UCNP‐sAv) and biotinylated QDs (biot‐QD) as acceptors.^[^ ^31^ ^]^ Without biotin in the sample, biot‐QDs bound to UCNP‐sAv via the strong biot‐sAv interaction (Figure 3a). Owing to the proximity between UCNP and QDs, excitation at 980 nm resulted in the typical Er^3+^ UC emission in the green (around 510 to 560 nm) and the red (around 640 to 680 nm) or FRET from UCNP‐to‐QD (due to the spectral overlap of green UCNP emission and QD absorption) followed by QD luminescence around 605 nm (Figure 3b). The FRET efficiency was estimated to be ca. 7% using excited‐state lifetimes of the UCNP donor, and it was assumed that only Er^3+^ ions close to the UCNP surface could participate in FRET. Addition of free biotin (the analytical target) to the UCNP‐sAv‐biot‐QD system resulted in competitive binding of biot‐QD and free biotin to UCNP‐sAv. Therefore, FRET and concomitantly the QD emission at 605 nm decreased with increasing biotin concentration (Figure 3c). Depending on the UCNP‐sAv and biot‐QD concentrations used in the assay, different nanomolar concentrations of biotin could be quantified with LODs down to ca. 5 nM.

An assay consisting of the NaYF_4_:Yb^3+^/Tm^3+^ UCNPs as FRET donors and CdTe QDs as acceptors was used to sense the Pb^2+^ ions.^[^ ^151^ ^]^ The UCNPs were modified with cationic polyetherimide (PEI), whereas the QDs were capped with anionic thioglycolic acid (TGA), such that the donor–acceptor pair could be assembled via electrostatic interaction. FRET was accomplished due to an overlap of the absorption band of the QDs and the Tm^3+^:^1^D_2_–^3^F_4_ (460 nm), Tm^3+^:^1^G_4_–^3^H_6_ (480 nm) emission bands of the UCNPs. Thus, under 980 nm, both Tm^3+^ and QD emission were observed. A Tm^3+^ luminescence lifetime change from 263 to 209 µs resulted in an *E*_FRET_ value of ∼21%. Introduction of Pb^2+^ (analyte) ions led to a noticeable decrease in the emission intensity of QDs due to the interaction of Pb^2+^ ions with the TGA on the surface of QDs. In serum, Pb^2+^ concentration‐dependent FRET could be used to quantify Pb^2+^ in a range of 20–3600 nM with an LOD of ca. 80 nM. A similar study applied NaYF_4_:Yb^3+^,Er^3+^UCNP to CdTe QDs FRET to detect Hg^2+^ ions.^[^ ^152^ ^]^ The surface of the UCNPs was modified with PEI allowing carboxyl‐capped QDs to adhere to their surface. Two types of QDs with excitonic peaks at 547 and 465 nm were studied, and only the first one was suitable as a FRET acceptor due to an overlap of its absorption band with the ^2^S_3/2_–^4^I_15/2_ Er^3+^ emission band (around 550 nm) in the UCNPs. Under 980 nm excitation, five emission bands were observed—four originating from UCNPs (around 410, 525, 550, and 660 nm) and one around 580 nm originating from QDs due to UCNP‐to‐QD FRET. Introduction of the QDs led to a change in the decay rate of the ^2^S_3/2_–^4^I_15/2_ radiative transition of the Er^3+^ ions from 0.56 to 0.78 µs^−1^ and thus, an *E*_FRET_ of approximately 28%. Addition of Hg^2+^ (analyte) ions led to the change of the QD surface states due to interaction between Hg^2+^ and carboxyl on the surface of QDs, which caused significant Hg^2+^ concentration‐dependent quenching of QD emission. Quantification was performed in different concentrations (0.1 and 0.15 g mL^−1^) of human serum with estimated LODs for Hg^2+^ of 15 and 17 nM, respectively.

A UCNP‐to‐QD FRET DNA assay was developed using o‐phosphorylethanolamine (PEA)‐capped NaYF_4_:Tm^3+^,Yb^3+^@NaYF_4_ core/shell UCNPs immobilized on aldehyde‐modified cellulose paper.^[^ ^165^ ^]^ The UCNPs were decorated with DNA probes to hybridize to HPRT1 gene fragments, which brought QDs conjugated with reporter DNA in close proximity by hybridizing to the UCNP‐DNA for FRET. Under 980 nm excitation, an emission band of the QDs with its maximum at 575 nm was observed. When QDs were not augmented with the HPRT1 target, the FRET ratio (and thus QD emission) was relatively low. Addition of the HPRT1 gene fragments to the QDs led to their adhesion to the PEA‐UCNPs, bringing donors and acceptors close enough to observe efficient FRET. This approach allowed detecting unlabeled oligonucleotide targets with an LOD down to 13 fmol.

NaYF_4_:Yb^3+^, Er^3+^ UCNPs to CdTe QD FRET was exploited for the development of a procalcitonin (PCT) immunoassay.^[^ ^166^ ^]^ The luminescence quantum yield of the UCNPs was estimated as 0.104% under 980 nm excitation. Combining this with the extinction coefficient of the QDs resulted in an *R*_0_ of ca. 3.2 nm. To realize the assay, the UCNPs were first coated with polyacrylic acid, and then 3D3 monoclonal antibodies were conjugated to the polymer‐coated UCNPs. CdTe QDs were coated with 3‐mercaptopropionic acid (MPA) and then bioconjugated with 2E6 monoclonal antibodies. The UCNP_D_‐3D3‐PCT‐2E6‐QD_A_ sandwich immunoassay resulted in UCNP‐to‐QD FRET and concomitant UCNP luminescence quenching with a maximum *E*_FRET_ of ∼33% and an estimated donor–acceptor distance of 3.6 nm.

A matrix metalloproteinase (MMP2) assay was designed using core‐shell UCNPs composed of NaYF_4_:Tm/Yb@NaYF_4_:Nd/Yb coated with silica functionalized with an MMP‐sensitive peptide with a cysteine residue.^[^ ^167^ ^]^ CuInS_2_/ZnS core‐shell QDs were subsequently conjugated to the thiolated peptide on the UCNPs. As a result, under 808 nm excitation, several Tm^3+^ emission bands (^4^I_6_–^3^F_4_ (390 nm), ^1^D_2_–^3^H_6_ (410 nm), ^1^D_2_–^3^F_4_ (450 nm), and ^1^G_4_–^3^H_6_ (470 nm)) and broad QD emission (500–750 nm) were observable. Introduction of MMP resulted in peptide digestion and concomitant FRET interruption, which led to a simultaneous decrease in the QD and increase in the UCNP emission within a 10^−5^ to 10^−2^ pg mL^−1^ MMP2 concentration range. The UCNP‐to‐QD FRET sensor was also employed in both cell and mouse models.

Although a translation into actual biosensing applications still needs to be demonstrated, various UCNP‐to‐QD FRET proof‐of‐concept systems have been successfully established. The NIR excitation of UCNPs combined with the color tuning of QDs remains a unique property of UCNP‐to‐QD FRET, and the recent advances in material research, such as smaller and brighter UCNPs, may lead to new avenues for translating UCNP‐to‐QD FRET into biosensing applications that are not accessible via other FRET systems. The high photostabilities of UCNPs and QDs may also result in FRET sensing applications under extreme environmental conditions, and the long luminescence lifetimes of UCNPs combined with the color multiplexing capabilities of both UCNPs and QDs are also attractive properties that have not been exploited in UCNP‐to‐QD FRET to date.

### 6.3 FRET Between FONs

**Figure 4:**
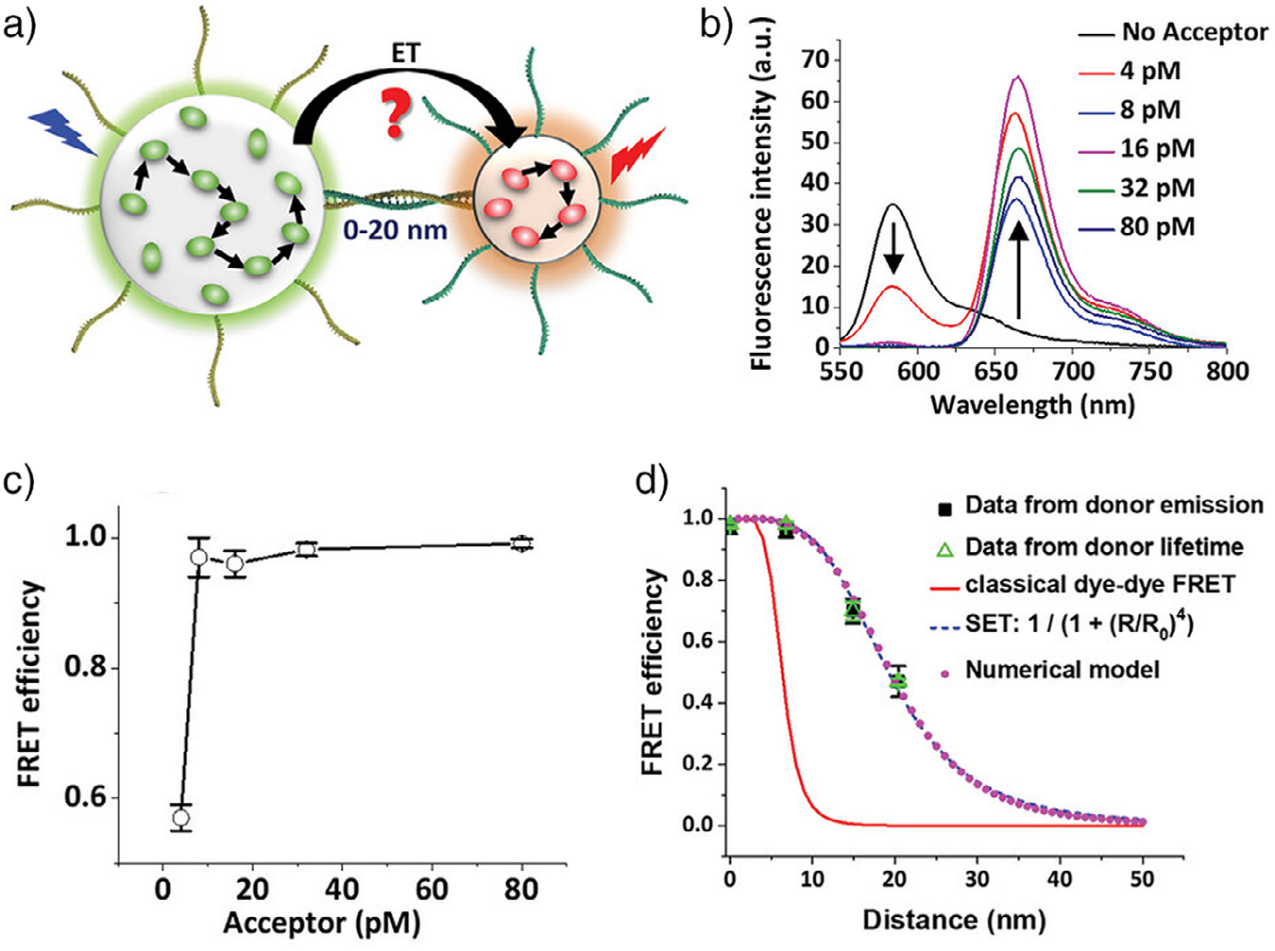
a) Donor (green) and acceptor (red) dye‐doped NPs composed of polymethacrylate derivatives loaded with either a bulky rhodamine derivative (donor) or a bulky Atto647N derivative (acceptor). For DNA hybridization, the NPs were functionalized with complementary oligonucleotides, which determined the FON–FON distance. b) Fluorescence spectra and c) FRET efficiencies of the NP FRET pairs using donor NPs (585 nm) with varying concentrations of the acceptor NPs (668 nm) for an NP–NP distance of 6.8 nm. d) The FRET efficiency showed an R_DA_^−4^ distance dependence. Reproduced with permission from Ref. [32], Copyright 2023 Wiley‐VCH GmbH.

FON–FON FRET is a younger research field compared to QD–QD or UCNP‐QD FRET. In contrast to QDs and UCNPs, most FONs are directly produced as water‐soluble NPs, especially in the case of core‐shell FONs because their core is made of hydrophobic polymers, which collapse in water to form the NPs. Thus, FON–FON FRET is an intrinsic water‐based problem. Arguably the first example of FON–FON FRET was reported in 2015 by Wu et al.^[^ ^168^ ^]^ They used Pdots of ∼95 nm diameter emitting around 450 and 550 nm. The Pdots were coated with polycationic polymers, which caused Pdot aggregation and concomitant FRET. Addition of DNA resulted in interaction with the oppositely charged polymers and dispersion of the Pdots, leading to decreased FRET. This deaggregation‐based FRET assay could detect DNA at very low concentrations (∼10^−14 ^M) but was not selective to a specific DNA sequence, which severely limited its use for actual DNA sensing. In a more recent example (Figure 4), Biswas et al. developed a FON–FON FRET DNA‐hybridization assay based on dye‐doped polymeric NPs of around 40 nm for the donor (doped with rhodamine B‐C_18_, λ_em_ = 585 nm) and 25 nm for the acceptor (doped with ATTO647‐C_18_, or Cy5‐C_18_, λ_em_ ∼670 nm).^[^ ^32^ ^]^ The surfaces of the two different FONs were functionalized with complementary DNA strands, which resulted in hybridization and brought the FONs in proximity for FRET. Despite the relatively large sizes of those FONs, the intra‐NP dye–dye energy migration resulted in very efficient sensitization of acceptor emission upon donor excitation with FRET efficiencies reaching up to 97%. Contrary to the *R*_DA_^−6^ distance dependence of FRET, the energy transfer followed the *R*_DA_^−4^ distance dependence of NSET, which could be related to the many possible donor–acceptor pairs, such that a simple point dipole–point dipole interaction would not apply anymore. The DNA hybridization could be quantified at picomolar concentrations.

FRET between water‐soluble Cdots (∼7 nm) immobilized in a mesoporous Al_2_O_3_ matrix was applied to sense oxygen.^[^ ^153^ ^]^ The blue Cdot donors (λ_em_ = 417 nm, Φ_f_ = 32%) with an exceptionally long luminescence lifetime (τ = 98 ns) could transfer energy to the red Cdot acceptors (λ_em_ = 630 nm, Φ_f_ = 28%) with *E*_FRET_ around 61% for a mass donor/acceptor ratio of 1/4. Upon oxygen penetration into the matrix, the red Cdots oxidized and their fluorescence intensity decreased, resulting in an oxygen concentration‐dependent change of the donor/acceptor fluorescence intensity ratio. The ratiometric FON–FON FRET sensor showed good selectivity for oxygen and short response and regeneration times of the mesoporous Al_2_O_3_ matrix. Other pairs of blue and red carbon‐based nanomaterials were able to undergo FRET and sense heavy metals.^[^ ^150^ ^]^ These above examples did not rely on the change in FRET rate for the detection. Instead, the metal ions introduced an additional energy loss channel, thus affecting the emission intensity of only one of the fluorophores in the assay without disrupting the FRET process. This allowed the ratio between the two emission bands to be used to quantify the amount of analyte. A recent example demonstrated FRET between Cdots to detect PCT.^[^ ^169^ ^]^ A blue Cdot donor was conjugated to a primary anti‐PCT antibody and a green Cdot acceptor to a secondary anti‐PCT antibody. Both antibody‐labeled Cdots specifically recognized PCT, bringing the donor and acceptor into close proximity upon antigen binding. This proximity enabled FRET and allowed sensitive and ratiometric detection of PCT in the 0.5–5 ng mL^−1^ range.

Despite their relatively large sizes, efficient energy migration in polymer FON donors can transport excitons close to the surface, where they become available for FRET. Whereas it seems more reasonable to simply use molecular acceptors on the donor FON surface, a few studies have shown that FON–FON FRET is possible. To evaluate the full potential of FON–FON FRET for biosensing, more systematic investigations concerning the energy transfer mechanism are required. An interesting finding was the *R*_DA_^−4^ distance dependence, which could be an actual advantage because longer distances may become accessible. Thus, further investigating the distance dependence with other FON systems could be of potential interest for future research. As FON–FON FRET is still in the early stages of fundamental understanding, there is much room for advancing the field and helping to discover the sweet spots of FON–FON FRET for biosensing.

### 6.4 Miscellaneous Inter‐NP FRET

**Figure 5:**
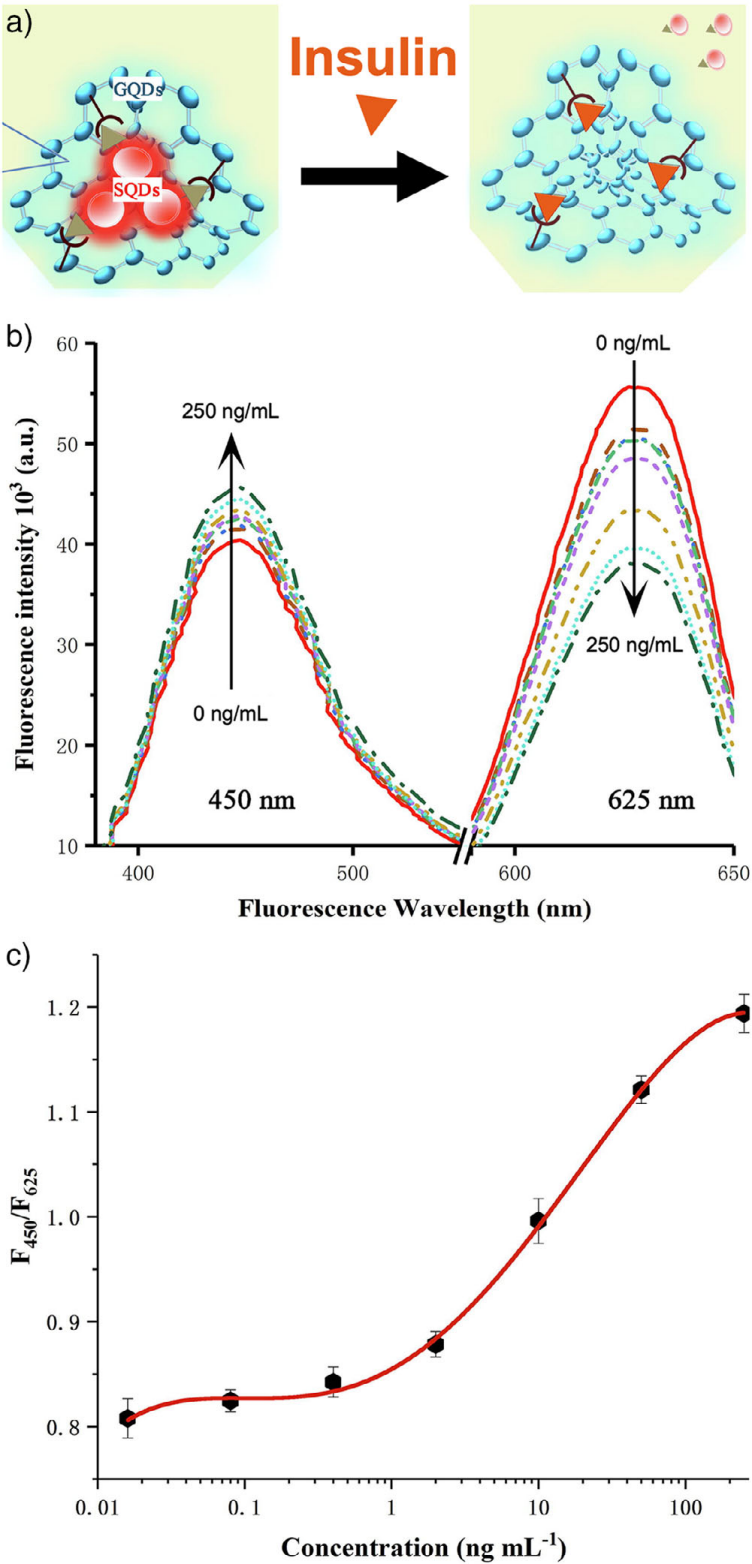
a) Competitive insulin assay principle using graphene QDs (gQDs, cyan) as donors and semiconductor QDs (QDs, red) as acceptors. gQDs were labeled with antibodies and QDs with antigen (insulin), which resulted in antibody‐antigen binding and FRET complex formation (left). Addition of insulin results in competitive binding and QD displacement from the gQDs (right). b) Fluorescence spectra of the probe (450 nm emission of gQDs and 625 nm emission of QDs) with varying concentrations of added insulin. c) Insulin assay calibration curve showing the donor/acceptor fluorescence intensity ratio over insulin concentration. Adapted with permission from Ref. [170], Copyright 2022 Elsevier.

Inter‐NP FRET has also been studied with other NP combinations, such as UCNP donors with CH_3_NH_3_PbBr_3_ perovskite NP acceptors,^[^ ^208^ ^]^ silica NP donors with gQD acceptors,^[^ ^209^ ^]^ QD donors and FON acceptors,^[^ ^210^, ^211^ ^]^ and polymer FON,^[^ ^212^ ^]^ Cdot,^[^ ^149^ ^]^ Pdot^[^ ^112^ ^]^ or gQD donors^[^ ^170^ ^]^ with QD acceptors. Only five of those pairs were dispersible in aqueous media, and three were used for FRET‐based biosensing or bioimaging. In the first example, Cdot donors (λ_em_ = 430 nm) and water‐soluble CdTe QD acceptors (λ_em_ = 570 nm) were associated, and a maximum *E*_FRET_ value of 57% was reported^[^ ^149^ ^]^ for a system with the following concentrations: Cdots: 2.8 × 10^−5^ mol L^−1^; CdTe: 2.0 × 10^−5^ mol L^−1^. The authors claimed that the addition of the chlortoluron led to selective quenching of the CdTe QD emission due to a strong interaction, while the Cdots remained unaffected. Although the study did not provide a detailed analysis of the interactions or photophysical phenomenon, a chlortoluron LOD of 7.8 × 10^−11 ^M was reported based on the evolution of the emission intensity of each NP with the change in the analyte concentration. Hybrids of poly[9,9‐dioctylfluorenyl‐2,7‐diyl)‐co‐4,7‐benzo{2,1,3}‐thiadiazole)] Pdots (donors) and three commercial QDs (acceptors) emitting around 655, 705, and 800 nm, respectively, were obtained by co‐precipitation of Pdots and QDs in the presence of amphiphilic copolymers and small thiolated ligands.^[^ ^112^ ^]^ The hybrids had a diameter of approximately 25 nm and could be conjugated with anti‐α‐tubulin antibodies for targeted imaging of microtubules in HeLa cells. An insulin assay was developed using gQDs (λ_em_ ∼450 nm, quantum yield ∼7%, diameter ∼10 nm) bioconjugated with monoclonal anti‐insulin antibodies as donors and CdTe QDs (λ_em_ ∼625 nm, quantum yield ∼4%, diameter ∼5 nm) coated with insulin as acceptors (Figure 5).^[^ ^170^ ^]^ The antibody‐insulin binding resulted in gQD‐to‐QD FRET (*E*_FRET_ = 41%) and dual (450 and 625 nm) emission. When insulin was added to the formed complexes, competitive binding dissociated the gQD antibodies and insulin QDs and reduced FRET, such that the donor/acceptor fluorescence intensity ratio increased with increasing insulin concentration. This competitive assay could quantify insulin in a concentration range from ca. 0.03 to 250 ng mL^−1^ in phosphate‐buffered saline and was also applied for insulin quantification in human serum samples with similar results to those obtained with enzyme‐linked immunosorbent assays (ELISA).

## 7 Summary and Outlook

While luminescent NPs have been widely used as FRET donors and acceptors in combination with molecular chromophores or fluorescent proteins and as NSET donors in combination with AuNPs, their pairing with other luminescent NPs has remained less common. Despite the unique and very advantageous photophysical properties of luminescent NPs, the distance restrictions imposed by FRET—which decrease the FRET efficiency with an inverse 6^th^ power of the donor–acceptor distance—are highly significant and cannot always be overcome. Designing and carefully characterizing such FRET systems is both difficult and time‐consuming and may not always lead to an actual improvement of analytical performance. However, providing a fundamental understanding has always been the first important step to accomplish useful applications. It is reasonable that the first optimization steps for luminescent NPs toward biosensors concern brightness, stability, and surface functionalization, whereas FRET and the required NP optimization for compactness have second priority. Therefore, it is not surprising that the most mature and smallest NPs, i.e., QDs, and, in particular, small CdTe QDs, have been more frequently used for inter‐NP FRET biosensing studies. Of the 25 examples discussed here (Table 2), 50% investigated QD‐QD FRET, 25% UCNP‐QD FRET, 20% FON–FON FRET, and less than 10% hybrids such as FON‐QD, which means that ca. 80% involved QDs.

Regardless of the importance of the NP diameters (which can be up to 10 times larger than *R*_0_) and the distance limitations, the feasibility of inter‐NP FRET has been clearly demonstrated, with some studies having found FRET efficiencies of up to 90% (Table 2).^[^ ^32^, ^158^, ^167^ ^]^ Considering that more compact NPs with higher brightness per volume values have only recently become a focus in NP design and synthesis, more inter‐NP FRET research can be expected in the future, and more inter‐NP FRET systems will become useful and beneficial for biosensing. Organic/inorganic hybrid systems can possibly offer a powerful strategy for exploiting the complementary properties of different types of NPs. These include i) high molar absorption coefficients and brightness of QDs, which can help achieve more efficient excitation and energy transfer; ii) narrow emission spectra, long luminescent lifetimes, and NIR‐excitation of the UCNPs, allowing both spectral and temporal multiplexing as well as high signal‐to‐noise ratios; and iii) efficient energy migration in FONs, which can mitigate the size effects of the luminescent NPs. Combining those unique photophysical characteristics with smaller NP sizes is clearly the key toward efficient NP‐based FRET biosensing systems, which will be challenging to design but potentially provide important advantages for bioanalytical applications. Higher‐order multiplexing (both spectral and temporal), lower limits of detection (with brighter NPs being detectable at lower concentrations), longer detection times (with stable emission intensities), enhanced biological interactions (on NP surfaces), and unique FRET cascade capabilities (inside and outside of the NPs) are very desirable features for biosensing and bioimaging, and luminescent NPs have the potential to combine all of them. While FRET systems with a single luminescent NP (as donor or acceptor) will likely remain dominant, inter‐NP FRET systems that combine the unique functional sweet spots of multiple NP types will certainly be able to provide special niche applications with the possibility to break the limits of analytical performance that cannot be broken otherwise.

## Conflict of Interests

The authors declare no conflict of interest.

## Data Availability

Data sharing is not applicable to this article as no new data were created or analyzed in this study.

## References

[1] T. Förster , Ann. Phys. 1948, 437, 55–75.

[2] F. Eckart , H.‐G. Löhmannsröben , N. Hildebrandt , Angew. Chem., Int. Ed. 2025, 64, e202416843. 10.1002/anie.202416843 40525781

[3] I. Medintz , N. Hildebrandt , FRET – Förster Resonance Energy Transfer, Wiley‐VCH Verlag GmbH & Co. KGaA, Weinheim, Germany 2014.

[4] B. Hellenkamp , S. Schmid , O. Doroshenko , O. Opanasyuk , R. Kühnemuth , S. Rezaei Adariani , B. Ambrose , M. Aznauryan , A. Barth , V. Birkedal , M. E. Bowen , H. Chen , T. Cordes , T. Eilert , C. Fijen , C. Gebhardt , M. Götz , G. Gouridis , E. Gratton , T. Ha , P. Hao , C. A. Hanke , A. Hartmann , J. Hendrix , L. L. Hildebrandt , V. Hirschfeld , J. Hohlbein , B. Hua , C. G. Hübner , E. Kallis , et al. Nat. Methods 2018, 15, 669–676. 30171252 10.1038/s41592-018-0085-0 PMC6121742

[5] G. Agam , C. Gebhardt , M. Popara , R. Mächtel , J. Folz , B. Ambrose , N. Chamachi , S. Y. Chung , T. D. Craggs , M. de Boer , D. Grohmann , T. Ha , A. Hartmann , J. Hendrix , V. Hirschfeld , C. G. Hübner , T. Hugel , D. Kammerer , H.‐S. Kang , A. N. Kapanidis , G. Krainer , K. Kramm , E. A. Lemke , E. Lerner , E. Margeat , K. Martens , J. Michaelis , J. Mitra , G. G. Moya Muñoz , R. B. Quast , et al. Nat. Methods 2023, 20, 523–535. 36973549 10.1038/s41592-023-01807-0 PMC10089922

[6] W. R. Algar , N. Hildebrandt , S. S. Vogel , I. L. Medintz , Nat. Methods 2019, 16, 815–829. 31471616 10.1038/s41592-019-0530-8

[7] L. Stryer , R. P. Haugland , Proc. Natl. Acad. Sci. USA 1967, 58, 719–726. 5233469 10.1073/pnas.58.2.719 PMC335693

[8] T. Ha , T. Enderle , D. F. Ogletree , D. S. Chemla , P. R. Selvin , S. Weiss , Proc. Natl. Acad. Sci. 1996, 93, 6264–6268. 8692803 10.1073/pnas.93.13.6264 PMC39010

[9] R. Roy , S. Hohng , T. Ha , Nat. Methods 2008, 5, 507–516. 18511918 10.1038/nmeth.1208 PMC3769523

[10] P. G. Wu , L. Brand , Anal. Biochem. 1994, 218, 1–13. 8053542 10.1006/abio.1994.1134

[11] L. Wu , C. Huang , B. P. Emery , A. C. Sedgwick , S. D. Bull , X.‐P. He , H. Tian , J. Yoon , J. L. Sessler , T. D. James , Chem. Soc. Rev. 2020, 49, 5110–5139. 32697225 10.1039/c9cs00318e PMC7408345

[12] J. B. Grimm , L. D. Lavis , Nat. Methods 2022, 19, 149–158. 34949811 10.1038/s41592-021-01338-6

[13] S. Tyagi , F. R. Kramer , Nat. Biotechnol. 1996, 14, 303–308. 9630890 10.1038/nbt0396-303

[14] K. Quan , C. Yi , X. Yang , X. He , J. Huang , K. Wang , TrAC Trends Anal. Chem. 2020, 124, 115784.

[15] A. Miyawaki , J. Llopis , R. Heim , J. M. McCaffery , J. A. Adams , M. Ikura , R. Y. Tsien , Nature 1997, 388, 882–887. 9278050 10.1038/42264

[16] M. Wang , Y. Da , Y. Tian , Chem. Soc. Rev. 2023, 52, 1189–1214. 36722390 10.1039/d2cs00419d

[17] K. E. Sapsford , B. Wildt , A. Mariani , A. B. Yeatts , I. Medintz , in FRET – Förster Resonance Energy Transfer, Wiley‐VCH Verlag GmbH & Co. KGaA, Weinheim, Germany, 2014, pp. 165–268.

[18] W. R. Algar , M. Massey , K. Rees , R. Higgins , K. D. Krause , G. H. Darwish , W. J. Peveler , Z. Xiao , H.‐Y. Tsai , R. Gupta , K. Lix , M. V. Tran , H. Kim , Chem. Rev. 2021, 121, 9243–9358. 34282906 10.1021/acs.chemrev.0c01176

[19] N. Hildebrandt , C. M. Spillmann , W. R. Algar , T. Pons , M. H. Stewart , E. Oh , K. Susumu , S. A. Díaz , J. B. Delehanty , I. L. Medintz , Chem. Rev. 2017, 117, 536–711. 27359326 10.1021/acs.chemrev.6b00030

[20] W. R. Algar , A. Szwarczewski , M. Massey , Anal. Chem. 2023, 95, 551–559. 36595310 10.1021/acs.analchem.2c03751

[21] N. Hildebrandt , M. Lim , N. Kim , D. Y. Choi , J.‐M. Nam , Chem. Commun. 2023, 59, 2352–2380. 10.1039/d2cc06178c 36727288

[22] C. Chen , N. Hildebrandt , TrAC Trends Anal. Chem. 2020, 123, 115748.

[23] C. Wu , B. Bull , C. Szymanski , K. Christensen , J. McNeill , ACS Nano 2008, 2, 2415–2423. 19206410 10.1021/nn800590n PMC2654197

[24] A. Reisch , A. S. Klymchenko , Small 2016, 12, 1968–1992. 26901678 10.1002/smll.201503396 PMC5405874

[25] Y. Wang , A. Hu , J. Mater. Chem. C 2014, 2, 6921.

[26] N. Hildebrandt , in FRET – Förster Resonance Energy Transfer, Wiley‐VCH Verlag GmbH & Co. KGaA, Weinheim, Germany 2014, pp. 105–163.

[27] N. Hildebrandt , K. D. Wegner , W. R. Algar , Coord. Chem. Rev. 2014, 273–274, 125–138.

[28] S. Bhuckory , E. Hemmer , Y.‐T. Wu , A. Yahia‐Ammar , F. Vetrone , N. Hildebrandt , Eur. J. Inorg. Chem. 2017, 2017, 5186–5195.

[29] B. Andreiuk , A. Reisch , M. Lindecker , G. Follain , N. Peyriéras , J. G. Goetz , A. S. Klymchenko , Small 2017, 13, 1701582. 10.1002/smll.201701582 28791769

[30] M. Chern , R. Toufanian , A. M. Dennis , Analyst 2020, 145, 5754–5767. 32715305 10.1039/d0an00746c PMC8275315

[31] L. Mattsson , K. D. Wegner , N. Hildebrandt , T. Soukka , RSC Adv. 2015, 5, 13270–13277.

[32] D. S. Biswas , P. Gaki , E. Cruz Da Silva , A. Combes , A. Reisch , P. Didier , A. S. Klymchenko , Adv. Mater. 2023, 35, 2301402. 10.1002/adma.202301402 37073109

[33] L. D. Lavis , R. T. Raines , ACS Chem. Biol. 2008, 3, 142–155. 18355003 10.1021/cb700248m PMC2802578

[34] A. H. Ashoka , I. O. Aparin , A. Reisch , A. S. Klymchenko , Chem. Soc. Rev. 2023, 52, 4525–4548. 37338018 10.1039/d2cs00464j PMC10351213

[35] C. Grazon , M. Chern , P. Lally , R. C. Baer , A. Fan , S. Lecommandoux , C. Klapperich , A. M. Dennis , J. E. Galagan , M. W. Grinstaff , Chem. Sci. 2022, 13, 6715–6731. 35756504 10.1039/d1sc06921g PMC9172442

[36] L. Kokko , K. Sandberg , T. Lövgren , T. Soukka , Anal. Chim. Acta 2004, 503, 155–162.

[37] J. E. Galagan , A. M. Dennis , C. Klapperich , M. W. Grinstaff , T. Nguyen , R. C. Baer , U. Kuzmanović , M. Zamani , M. Chen , M. Chern , C. Grazon , Microbial Based Biosensors 2019, United States Patent, US20190195894A1.

[38] V. Parthasarathy , S. Fery‐Forgues , E. Campioli , G. Recher , F. Terenziani , M. Blanchard‐Desce , Small 2011, 7, 3219–3229. 21972222 10.1002/smll.201100726

[39] R. Gupta , W. J. Peveler , K. Lix , W. R. Algar , Anal. Chem. 2019, 91, 10955–10960. 31403282 10.1021/acs.analchem.9b02881

[40] K. Lix , K. D. Krause , H. Kim , W. R. Algar , J. Phys. Chem. C 2020, 124, 17387–17400.

[41] S. Wen , J. Zhou , K. Zheng , A. Bednarkiewicz , X. Liu , D. Jin , Nat. Commun. 2018, 9, 2415. 29925838 10.1038/s41467-018-04813-5 PMC6010470

[42] M. Quintanilla , E. Hemmer , J. Marques‐Hueso , S. Rohani , G. Lucchini , M. Wang , R. R. Zamani , V. Roddatis , A. Speghini , B. S. Richards , F. Vetrone , Nanoscale 2022, 14, 1492–1504. 35024718 10.1039/d1nr06319g

[43] F. Morgner , D. Geißler , S. Stufler , N. G. Butlin , H.‐G. Löhmannsröben , N. Hildebrandt , Angew. Chem., Int. Ed. 2010, 49, 7570–7574. 10.1002/anie.201002943 20806303

[44] K. Davis , B. Cole , M. Ghelardini , B. A. Powell , O. T. Mefford , Langmuir 2016, 32, 13716–13727. 27966977 10.1021/acs.langmuir.6b03644

[45] K. Binder , A. Milchev , J. Polym. Sci. Part B Polym. Phys. 2012, 50, 1515–1555.

[46] B. Hötzer , I. L. Medintz , N. Hildebrandt , Small 2012, 8, 2297–2326. 22678833 10.1002/smll.201200109

[47] M. Cardoso Dos Santos , W. R. Algar , I. L. Medintz , N. Hildebrandt , TrAC Trends Anal. Chem. 2020, 125, 115819.

[48] P. T. Snee , TrAC Trends Anal. Chem. 2020, 123, 115750.

[49] J. Shi , F. Tian , J. Lyu , M. Yang , J. Mater. Chem. B 2015, 3, 6989–7005. 32262700 10.1039/c5tb00885a

[50] A. R. Clapp , E. R. Goldman , H. Mattoussi , Nat. Protoc. 2006, 1, 1258–1266. 17406409 10.1038/nprot.2006.184

[51] B. O. Dabbousi , J. Rodriguez‐Viejo , F. V. Mikulec , J. R. Heine , H. Mattoussi , R. Ober , K. F. Jensen , M. G. Bawendi , J. Phys. Chem. B 1997, 101, 9463–9475.

[52] N. Zhan , G. Palui , H. Mattoussi , Nat. Protoc. 2015, 10, 859–874. 25974095 10.1038/nprot.2015.050

[53] M. Chern , T. T. Nguyen , A. H. Mahler , A. M. Dennis , Nanoscale 2017, 9, 16446–16458. 29063928 10.1039/c7nr04296e

[54] K. E. Sapsford , T. Pons , I. L. Medintz , S. Higashiya , F. M. Brunel , P. E. Dawson , H. Mattoussi , J. Phys. Chem. C 2007, 111, 11528–11538.

[55] R. Gill , M. Zayats , I. Willner , Angew. Chem., Int. Ed. 2008, 47, 7602–7625. 10.1002/anie.200800169 18810756

[56] J.‐C. G. Bünzli , Acc. Chem. Res. 2006, 39, 53–61. 16411740 10.1021/ar0400894

[57] J. G. Bünzli , Eur. J. Inorg. Chem. 2017, 2017, 5058–5063.

[58] M. Sy , A. Nonat , N. Hildebrandt , L. J. Charbonnière , Chem. Commun. 2016, 52, 5080–5095. 10.1039/c6cc00922k 26911318

[59] J.‐C. G. Bünzli , Coord. Chem. Rev. 2015, 293–294, 19–47.

[60] S. J. Strickler , R. A. Berg , J. Chem. Phys. 1962, 37, 814–822.

[61] M. V. DaCosta , S. Doughan , Y. Han , U. J. Krull , Anal. Chim. Acta 2014, 832, 1–33. 24890691 10.1016/j.aca.2014.04.030

[62] M. Wang , H. Ye , L. You , X. Chen , ACS Appl. Mater. Interfaces 2016, 8, 574–581. 26651854 10.1021/acsami.5b09607

[63] W. Zheng , S. Zhou , Z. Chen , P. Hu , Y. Liu , D. Tu , H. Zhu , R. Li , M. Huang , X. Chen , Angew. Chem., Int. Ed. 2013, 52, 6671–6676. 10.1002/anie.201302481 23658009

[64] Q. Ju , D. Tu , Y. Liu , R. Li , H. Zhu , J. Chen , Z. Chen , M. Huang , X. Chen , J. Am. Chem. Soc. 2012, 134, 1323–1330. 22145918 10.1021/ja2102604

[65] D. Tu , L. Liu , Q. Ju , Y. Liu , H. Zhu , R. Li , X. Chen , Angew. Chem., Int. Ed. 2011, 50, 6306–6310. 10.1002/anie.201100303 21612007

[66] C. Charpentier , V. Cifliku , J. Goetz , A. Nonat , C. Cheignon , M. Cardoso Dos Santos , L. Francés‐Soriano , K. Wong , L. J. Charbonnière , N. Hildebrandt , Chem. Eur. J. 2020, 26, 14602–14611. 32501573 10.1002/chem.202002007

[67] X. Wang , S. Niazi , H. Yukun , W. Sun , S. Wu , N. Duan , X. Hun , Z. Wang , Microchim. Acta 2017, 184, 4021–4027.

[68] J.‐Q. Gu , J. Shen , L.‐D. Sun , C.‐H. Yan , J. Phys. Chem. C 2008, 112, 6589–6593.

[69] Y. Tang , J. Hu , A. H. Elmenoufy , X. Yang , ACS Appl. Mater. Interfaces 2015, 7, 12261–12269. 25974980 10.1021/acsami.5b03067

[70] W. Zhang , X. Zhang , Y. Shen , F. Shi , C. Song , T. Liu , P. Gao , B. Lan , M. Liu , S. Wang , L. Fan , H. Lu , Biomaterials 2018, 184, 31–40. 30195803 10.1016/j.biomaterials.2018.09.001

[71] Y. Liu , Y. Lei , D. Li , G. Li , D. Li , Y. Li , Dyes Pigments 2024, 221, 111799.

[72] J. Gu , L. Sun , Z. Yan , C. Yan , Chem. Asian J. 2008, 3, 1857–1864. 18726878 10.1002/asia.200800230

[73] D. Casanova , D. Giaume , T. Gacoin , J.‐P. Boilot , A. Alexandrou , J. Phys. Chem. B 2006, 110, 19264–19270. 17004778 10.1021/jp063229v

[74] S. L. Yefimova , T. N. Tkacheva , P. O. Maksimchuk , I. I. Bespalova , K. O. Hubenko , V. K. Klochkov , A. V. Sorokin , Y. V. Malyukin , J. Lumin. 2017, 192, 975–981.

[75] J. Xu , L. Francés‐Soriano , J. Guo , T. Hallaj , X. Qiu , N. Hildebrandt , in Frontiers in Nanoscience, Elsevier, Amsterdam, The Netherlands, 2020, Vol. 16, pp. 25–65.

[76] F. E. Auzel , Proc. IEEE 1973, 61, 758–786.

[77] J. A. Curcio , C. C. Petty , J. Opt. Soc. Am. 1951, 41, 302.

[78] M. Kaiser , C. Würth , M. Kraft , T. Soukka , U. Resch‐Genger , Nano Res. 2019, 12, 1871–1879.

[79] M. Kaiser , C. Würth , M. Kraft , I. Hyppänen , T. Soukka , U. Resch‐Genger , Nanoscale 2017, 9, 10051–10058. 28686275 10.1039/c7nr02449e

[80] S. Bhuckory , S. Lahtinen , N. Höysniemi , J. Guo , X. Qiu , T. Soukka , N. Hildebrandt , Nano Lett. 2023, 23, 2253–2261. 36729707 10.1021/acs.nanolett.2c04899

[81] L. Francés‐Soriano , N. Estebanez , J. Pérez‐Prieto , N. Hildebrandt , Adv. Funct. Mater. 2022, 32, 2201541.

[82] L. Francés‐Soriano , N. Peruffo , M. M. Natile , N. Hildebrandt , Analyst 2020, 145, 2543–2553. 32043497 10.1039/c9an02532d

[83] F. Pini , L. Francés‐Soriano , N. Peruffo , A. Barbon , N. Hildebrandt , M. M. Natile , ACS Appl. Mater. Interfaces 2022, 14, 11883–11894. 35213132 10.1021/acsami.1c23498

[84] F. T. Rabouw , P. T. Prins , P. Villanueva‐Delgado , M. Castelijns , R. G. Geitenbeek , A. Meijerink , ACS Nano 2018, 12, 4812–4823. 29648802 10.1021/acsnano.8b01545 PMC5968434

[85] Y. Wang , K. Liu , X. Liu , K. Dohnalová , T. Gregorkiewicz , X. Kong , M. C. G. Aalders , W. J. Buma , H. Zhang , J. Phys. Chem. Lett. 2011, 2, 2083–2088.

[86] F. Pini , L. Francés‐Soriano , V. Andrigo , M. M. Natile , N. Hildebrandt , ACS Nano 2023, 17, 4971–4984. 36867492 10.1021/acsnano.2c12523

[87] H. S. Zhou , I. Honma , H. Komiyama , J. W. Haus , Phys. Rev. B 1994, 50, 12052–12056. 10.1103/physrevb.50.12052 9975346

[88] B. Jin , S. Wang , M. Lin , Y. Jin , S. Zhang , X. Cui , Y. Gong , A. Li , F. Xu , T. J. Lu , Biosens. Bioelectron. 2017, 90, 525–533. 27825886 10.1016/j.bios.2016.10.029

[89] X. Lao , Y. Liu , L. Li , M. Song , Y. Ma , M. Yang , G. Chen , J. Hao , Aggregate 2024, 5, e448.

[90] Y. Liu , Q. Ouyang , H. Li , M. Chen , Z. Zhang , Q. Chen , J. Agric. Food Chem. 2018, 66, 6188–6195. 29847117 10.1021/acs.jafc.8b00546

[91] Y. Ma , M. Song , L. Li , X. Lao , Y. Liu , M. Wong , M. Yang , H. Chen , J. Hao , Biosens. Bioelectron. 2024, 243, 115778. 10.1016/j.bios.2023.115778 39492185

[92] M. K. Abraham , A. S. Madanan , S. Varghese , A. I. Shkhair , G. Indongo , G. Rajeevan , N. S. Vijila , S. George , Analyst 2024, 149, 231–243. 10.1039/d3an01405c 38031450

[93] Z. Liu , C. Shang , H. Ma , M. You , Nanotechnology 2020, 31, 235501. 32069442 10.1088/1361-6528/ab776d

[94] S. G. Stenspil , J. Chen , M. B. Liisberg , A. H. Flood , B. W. Laursen , Chem. Sci. 2024, 15, 5531–5538. 38638234 10.1039/d3sc05496a PMC11023049

[95] K. Trofymchuk , A. Reisch , P. Didier , F. Fras , P. Gilliot , Y. Mely , A. S. Klymchenko , Nat. Photonics 2017, 11, 657–663. 28983324 10.1038/s41566-017-0001-7 PMC5624503

[96] L. Gartzia‐Rivero , L. Cerdán , J. Bañuelos , E. Enciso , Í. López Arbeloa , Á. Costela , I. García‐Moreno , J. Phys. Chem. C 2014, 118, 13107–13117.

[97] C. Martin , S. Bhattacharyya , A. Patra , A. Douhal , Photochem. Photobiol. Sci. 2014, 13, 1241–1252. 24969364 10.1039/c4pp00086b

[98] C. Wu , Y. Zheng , C. Szymanski , J. McNeill , J. Phys. Chem. C 2008, 112, 1772–1781. 10.1021/jp074149+ PMC2600541 19221582

[99] E. Campioli , C. Rouxel , M. Campanini , L. Nasi , M. Blanchard‐Desce , F. Terenziani , Small 2013, 9, 1982–1988. 23292762 10.1002/smll.201202504

[100] S. G. Stenspil , B. W. Laursen , Chem. Sci. 2024, 15, 8625–8638. 38873083 10.1039/d4sc01352b PMC11168078

[101] Y. Tamai , H. Ohkita , H. Benten , S. Ito , J. Phys. Chem. Lett. 2015, 6, 3417–3428. 26269208 10.1021/acs.jpclett.5b01147

[102] A. M. Gharbi , D. S. Biswas , O. Crégut , P. Malý , P. Didier , A. Klymchenko , J. Léonard , Nanoscale 2024, 16, 11550–11563. 38868990 10.1039/d4nr00325j

[103] C. Grazon , J. Rieger , B. Charleux , G. Clavier , R. Méallet‐Renault , J. Phys. Chem. C 2014, 118, 13945–13952.

[104] N. Melnychuk , A. S. Klymchenko , J. Am. Chem. Soc. 2018, 140, 10856–10865. 30067022 10.1021/jacs.8b05840

[105] L. Kacenauskaite , S. G. Stenspil , A. H. Olsson , A. H. Flood , B. W. Laursen , J. Am. Chem. Soc. 2022, 144, 19981–19989. 36256621 10.1021/jacs.2c08540

[106] T. M. Swager , Acc. Chem. Res. 1998, 31, 201–207.

[107] S. W. Thomas , G. D. Joly , T. M. Swager , Chem. Rev. 2007, 107, 1339–1386. 17385926 10.1021/cr0501339

[108] Y. Wu , C. Shi , G. Wang , H. Sun , S. Yin , J. Mater. Chem. B 2022, 10, 2995–3015. 35393982 10.1039/d1tb02816b

[109] J. Yu , Y. Rong , C.‐T. Kuo , X.‐H. Zhou , D. T. Chiu , Anal. Chem. 2017, 89, 42–56. 28105818 10.1021/acs.analchem.6b04672 PMC5682631

[110] C. Wu , D. T. Chiu , Angew. Chem., Int. Ed. 2013, 52, 3086–3109. 10.1002/anie.201205133 PMC5616106 23307291

[111] Y. Han , T. Chen , Y. Li , L. Chen , L. Wei , L. Xiao , Anal. Chem. 2019, 91, 11146–11153. 31402640 10.1021/acs.analchem.9b01849

[112] Y.‐H. Chan , F. Ye , M. E. Gallina , X. Zhang , Y. Jin , I.‐C. Wu , D. T. Chiu , J. Am. Chem. Soc. 2012, 134, 7309–7312. 22515545 10.1021/ja3022973 PMC3350096

[113] S. Ding , A. A. Cargill , S. R. Das , I. L. Medintz , J. C. Claussen , Sensors 2015, 15, 14766–14787. 26110411 10.3390/s150614766 PMC4507682

[114] K. Pu , A. J. Shuhendler , J. Rao , Angew. Chem., Int. Ed. 2013, 52, 10325–10329. 10.1002/anie.201303420 PMC4079533 23943508

[115] S. Grigalevicius , M. Forster , S. Ellinger , K. Landfester , U. Scherf , Macromol. Rapid Commun. 2006, 27, 200–202.

[116] R. A. Ponzio , R. M. Spada , A. B. Wendel , M. V. Forcone , F. D. Stefani , C. A. Chesta , R. E. Palacios , J. Phys. Chem. C 2021, 125, 23299–23312.

[117] K.‐Y. Pu , B. Liu , Adv. Funct. Mater. 2011, 21, 3408–3423.

[118] B. Wang , B. N. Queenan , S. Wang , K. P. R. Nilsson , G. C. Bazan , Adv. Mater. 2019, 31, 1806701. 10.1002/adma.201806701 30698856

[119] K.‐Y. Pu , Z. Luo , K. Li , J. Xie , B. Liu , J. Phys. Chem. C 2011, 115, 13069–13075.

[120] C. Fan , S. Wang , J. W. Hong , G. C. Bazan , K. W. Plaxco , A. J. Heeger , Proc. Natl. Acad. Sci. USA 2003, 100, 6297–6301. 12750470 10.1073/pnas.1132025100 PMC164440

[121] K.‐Y. Pu , K. Li , B. Liu , Adv. Mater. 2010, 22, 643–646. 20217765 10.1002/adma.200902409

[122] M. P. Robin , R. K. O'Reilly , Polym. Int. 2015, 64, 174–182.

[123] T. Yudhistira , E. C. Da Silva , A. Combes , M. Lehmann , A. Reisch , A. S. Klymchenko , Small Methods 2023, 7, 2201452. 10.1002/smtd.202201452 36808832

[124] C. Grazon , J. Rieger , R. Méallet‐Renault , G. Clavier , B. Charleux , Macromol. Rapid Commun. 2011, 32, 699–705. 21491536 10.1002/marc.201100008

[125] R. Méallet‐Renault , A. Hérault , J.‐J. Vachon , R. B. Pansu , S. Amigoni‐Gerbier , C. Larpent , Photochem. Photobiol. Sci. 2006, 5, 300–310. 16520865 10.1039/b513215k

[126] A. Reisch , P. Didier , L. Richert , S. Oncul , Y. Arntz , Y. Mély , A. S. Klymchenko , Nat. Commun. 2014, 5, 4089. 24909912 10.1038/ncomms5089

[127] A. M. Breul , M. D. Hager , U. S. Schubert , Chem. Soc. Rev. 2013, 42, 5366–5407. 23482971 10.1039/c3cs35478d

[128] C. Grazon , J. Rieger , R. Méallet‐Renault , B. Charleux , G. Clavier , Macromolecules 2013, 46, 5167–5176.

[129] G. Sun , M. Y. Berezin , J. Fan , H. Lee , J. Ma , K. Zhang , K. L. Wooley , S. Achilefu , Nanoscale 2010, 2, 548–558. 20644758 10.1039/b9nr00304e

[130] J. Liu , M. Evrard , X. Cai , G. Feng , N. Tomczak , L. G. Ng , B. Liu , J. Mater. Chem. B 2018, 6, 2630–2636. 32254481 10.1039/c8tb00386f

[131] W.‐C. Wu , C.‐Y. Chen , Y. Tian , S.‐H. Jang , Y. Hong , Y. Liu , R. Hu , B. Z. Tang , Y.‐T. Lee , C.‐T. Chen , W.‐C. Chen , A. K.‐Y. Jen , Adv. Funct. Mater. 2010, 20, 1413–1423.

[132] J. Yang , Z. Xu , L. Yu , B. Wang , R. Hu , J. Tang , J. Lv , H. Xiao , X. Tan , G. Wang , J.‐X. Li , Y. Liu , P.‐L. Shao , B. Zhang , Angew. Chem., Int. Ed. 2024, 63, e202318800. 10.1002/anie.202318800 38443316

[133] Y. Si , C. Grazon , G. Clavier , J.‐F. Audibert , B. Sclavi , R. Méallet‐Renault , Photochem. Photobiol. Sci. 2022, 21, 1249–1255. 35428949 10.1007/s43630-022-00215-1

[134] A. Nagai , R. Yoshii , T. Otsuka , K. Kokado , Y. Chujo , Langmuir 2010, 26, 15644–15649. 20815359 10.1021/la102597y

[135] M. Pan , G. M. Cruz , C. Grazon , D. Kechkeche , L. H. Renault , G. Clavier , R. Méallet‐Renault , ACS Appl. Polym. Mater. 2022, 4, 5482–5492.

[136] F. Arcudi , L. Đorđević , M. Prato , Acc. Chem. Res. 2019, 52, 2070–2079. 31335113 10.1021/acs.accounts.9b00249

[137] C. Ji , Y. Zhou , R. M. Leblanc , Z. Peng , ACS Sens. 2020, 5, 2724–2741. 32812427 10.1021/acssensors.0c01556

[138] F.‐T. Wang , L.‐N. Wang , J. Xu , K.‐J. Huang , X. Wu , Analyst 2021, 146, 4418–4435. 34195700 10.1039/d1an00466b

[139] S. Miao , K. Liang , B. Kong , Mater. Chem. Front. 2019, 4, 128–139.

[140] X. Fu , D. Gu , S. Zhao , N. Zhou , H. Zhang , J. Fluoresc. 2017, 27, 1597–1605. 28401410 10.1007/s10895-017-2095-1

[141] S. Mohammadi , A. Salimi , S. Hamd‐Ghadareh , F. Fathi , F. Soleimani , Anal. Biochem. 2018, 557, 18–26. 29908158 10.1016/j.ab.2018.06.008

[142] X. Shen , L. Xu , W. Zhu , B. Li , J. Hong , X. Zhou , New J. Chem. 2017, 41, 9230–9235.

[143] K. Mintz , E. Waidely , Y. Zhou , Z. Peng , A. O. Al‐Youbi , A. S. Bashammakh , M. S. El‐Shahawi , R. M. Leblanc , Anal. Chim. Acta 2018, 1041, 114–121. 30340683 10.1016/j.aca.2018.08.055

[144] J. S. Sidhu , A. Singh , N. Garg , N. Singh , ACS Appl. Mater. Interfaces 2017, 9, 25847–25856. 28737377 10.1021/acsami.7b07046

[145] M.‐J. Cho , S.‐Y. Park , Sens. Actuators, B 2019, 282, 719–729.

[146] Y. Kim , G. Jang , T. S. Lee , ACS Appl. Mater. Interfaces 2015, 7, 15649–15657. 26112227 10.1021/acsami.5b04724

[147] J. Hou , Q. Chen , X. Meng , D. Wang , H. Liu , W. Feng , Microchem. J. 2025, 208, 112576.

[148] W. Dong , R. Wang , X. Gong , W. Liang , C. Dong , Spectrochim. Acta. A. Mol. Biomol. Spectrosc. 2019, 213, 90–96. 30684884 10.1016/j.saa.2019.01.040

[149] H. Tao , X. Liao , C. Sun , X. Xie , F. Zhong , Z. Yi , Y. Huang , Spectrochim. Acta. A. Mol. Biomol. Spectrosc. 2015, 136, 1328–1334. 25456675 10.1016/j.saa.2014.10.020

[150] M. K. Chini , V. Kumar , A. Javed , S. Satapathi , Nano‐Struct. Nano‐Objects 2019, 19, 100347.

[151] S. Xu , S. Xu , Y. Zhu , W. Xu , P. Zhou , C. Zhou , B. Dong , H. Song , Nanoscale 2014, 6, 12573–12579. 25184968 10.1039/c4nr03092c

[152] S. Cui , S. Xu , H. Song , W. Xu , X. Chen , D. Zhou , Z. Yin , W. Han , RSC Adv. 2015, 5, 99099–99106.

[153] Y. He , J. He , L. Wang , Z. Yu , H. Zhang , Y. Liu , B. Lei , Sens. Actuators, B 2017, 251, 918–926.

[154] S. Wang , N. Mamedova , N. A. Kotov , W. Chen , J. Studer , Nano Lett. 2002, 2, 817–822.

[155] Q. Ma , X.‐G. Su , X.‐Y. Wang , Y. Wan , C.‐L. Wang , B. Yang , Q.‐H. Jin , Talanta 2005, 67, 1029–1034. 18970275 10.1016/j.talanta.2005.04.036

[156] C.‐Y. Chen , C.‐T. Cheng , C.‐W. Lai , P.‐W. Wu , K.‐C. Wu , P.‐T. Chou , Y.‐H. Chou , H.‐T. Chiu , Chem. Commun. 2006, 0, 263–265. 10.1039/b512677k 16391728

[157] Y. Li , Q. Ma , X. Wang , X. Su , Luminescence 2007, 22, 60–66. 17089351 10.1002/bio.927

[158] S. Mayilo , J. Hilhorst , A. S. Susha , C. Höhl , T. Franzl , T. A. Klar , A. L. Rogach , J. Feldmann , J. Phys. Chem. C 2008, 112, 14589–14594.

[159] T.‐C. Liu , H.‐L. Zhang , J.‐H. Wang , H.‐Q. Wang , Z.‐H. Zhang , X.‐F. Hua , Y.‐C. Cao , Q.‐M. Luo , Y.‐D. Zhao , Anal. Bioanal. Chem. 2008, 391, 2819–2824. 18537029 10.1007/s00216-008-2189-3

[160] T. Shiraki , Y. Tsuchiya , S. Shinkai , Chem. Lett. 2010, 39, 156–158. 10.1021/ja1067349 20836560

[161] U. O. S. Seker , T. Ozel , H. V. Demir , Nano Lett. 2011, 11, 1530–1539. 21428276 10.1021/nl104295b

[162] B.‐B. Wang , Q. Wang , Y.‐G. Jin , M.‐H. Ma , Z.‐X. Cai , J. Photochem. Photobiol. Chem. 2015, 299, 131–137.

[163] W. J. Peveler , H. Jia , T. Jeen , K. Rees , T. J. Macdonald , Z. Xia , W.‐I. Katherine Chio , S. Moorthy , I. P. Parkin , C. J. Carmalt , W. Russ Algar , T.‐C. Lee , Chem. Commun. 2019, 55, 5495–5498. 10.1039/c9cc00410f 31017133

[164] Y. S. Borghei , M. Hosseini , M. R. Ganjali , J. Photochem. Photobiol. Chem. 2020, 391, 112351.

[165] S. Doughan , U. Uddayasankar , U. J. Krull , Anal. Chim. Acta 2015, 878, 1–8. 26002323 10.1016/j.aca.2015.04.036

[166] Y. Zhou , X. Shao , Y. Han , H. Zhang , Anal. Methods 2018, 10, 1015–1022.

[167] Y.‐C. Chan , M.‐H. Chan , C.‐W. Chen , R.‐S. Liu , M. Hsiao , D. P. Tsai , ACS Omega 2018, 3, 1627–1634. 30023811 10.1021/acsomega.7b01494 PMC6045330

[168] M. Wu , X. Xu , J. Wang , L. Li , ACS Appl. Mater. Interfaces 2015, 7, 8243–8250. 25823879 10.1021/acsami.5b01338

[169] Y. Tian , Y. Du , Z. Chen , L. Li , R. Yan , X. Li , J. Yue , Spectrochim. Acta. A. Mol. Biomol. Spectrosc. 2025, 338, 126207. 40215845 10.1016/j.saa.2025.126207

[170] G. Yu , Z. Sun , Y. Wu , N. Sai , Spectrochim. Acta. A. Mol. Biomol. Spectrosc. 2022, 268, 120641. 34865977 10.1016/j.saa.2021.120641

[171] H. Xu , X. Huang , W. Zhang , G. Chen , W. Zhu , X. Zhong , ChemPhysChem 2010, 11, 3167–3171. 20872922 10.1002/cphc.201000287

[172] M. Werwie , X. Xu , M. Haase , T. Basché , H. Paulsen , Langmuir 2012, 28, 5810–5818. 22401299 10.1021/la204970a

[173] C. Chen , B. Corry , L. Huang , N. Hildebrandt , J. Am. Chem. Soc. 2019, 141, 11123–11141. 31251609 10.1021/jacs.9b03383

[174] M. Mubeen , M. A. Khalid , M. Mukhtar , P. Sumreen , T. Gul , N. ul Ain , S. Shahrum , M. Tabassum , A. Ul‐Hamid , A. Iqbal , Photochem. Photobiol. 2022, 98, 1017–1024. 35092012 10.1111/php.13599

[175] K. F. Chou , A. M. Dennis , Sensors 2015, 15, 13288–13325. 26057041 10.3390/s150613288 PMC4507609

[176] J. Hottechamps , T. Noblet , C. Méthivier , S. Boujday , L. Dreesen , Nanoscale 2023, 15, 2614–2623. 36648212 10.1039/d2nr06161a

[177] C. R. Kagan , C. B. Murray , M. Nirmal , M. G. Bawendi , Phys. Rev. Lett. 1996, 76, 1517–1520. 10061743 10.1103/PhysRevLett.76.1517

[178] H. Sugimoto , K. Furuta , M. Fujii , J. Phys. Chem. C 2016, 120, 24469–24475.

[179] R. Koole , P. Liljeroth , C. de Mello Donegá , D. Vanmaekelbergh , A. Meijerink , J. Am. Chem. Soc. 2006, 128, 10436–10441. 16895408 10.1021/ja061608w

[180] A. Wolf , V. Lesnyak , N. Gaponik , A. Eychmüller , J. Phys. Chem. Lett. 2012, 3, 2188–2193. 26295769 10.1021/jz300726n

[181] W. Yin , N. Kim , J. Jeong , K. S. Kim , H. Chae , T. K. Ahn , J. Phys. Chem. C 2017, 121, 4799–4805.

[182] M. Achermann , M. A. Petruska , S. A. Crooker , V. I. Klimov , J. Phys. Chem. B 2003, 107, 13782–13787.

[183] C.‐H. Wang , C.‐W. Chen , C.‐M. Wei , Y.‐F. Chen , C.‐W. Lai , M.‐L. Ho , P.‐T. Chou , J. Phys. Chem. C 2009, 113, 15548–15552.

[184] O. Erdem , K. Gungor , B. Guzelturk , I. Tanriover , M. Sak , M. Olutas , D. Dede , Y. Kelestemur , H. V. Demir , Nano Lett. 2019, 19, 4297–4305. 31185570 10.1021/acs.nanolett.9b00681

[185] C. Wang , E. A. Weiss , Nano Lett. 2017, 17, 5666–5671. 28786684 10.1021/acs.nanolett.7b02559

[186] S. Sarkar , A. R. Maity , N. S. Karan , N. Pradhan , J. Phys. Chem. C 2013, 117, 21988–21994.

[187] M. Noh , T. Kim , H. Lee , C.‐K. Kim , S.‐W. Joo , K. Lee , Colloids Surf. Physicochem. Eng. Asp. 2010, 359, 39–44.

[188] R. Osovsky , A. Shavel , N. Gaponik , L. Amirav , A. Eychmüller , H. Weller , E. Lifshitz , J. Phys. Chem. B 2005, 109, 20244–20250. 16853618 10.1021/jp0526795

[189] T. Noblet , J. Hottechamps , M. Erard , L. Dreesen , J. Phys. Chem. C 2022, 126, 15309–15318.

[190] L. Xu , J. Xu , W. Li , Z. Weiming , P. Sun , Z. Ma , X. Huang , K. Chen , J. Mater. Sci. 2007, 42, 9696–9699.

[191] R. Wargnier , A. V. Baranov , V. G. Maslov , V. Stsiapura , M. Artemyev , M. Pluot , A. Sukhanova , I. Nabiev , Nano Lett. 2004, 4, 451–457.

[192] P. Roy , G. Devatha , S. Roy , A. Rao , P. P. Pillai , J. Phys. Chem. Lett. 2020, 11, 5354–5360. 32539403 10.1021/acs.jpclett.0c01360

[193] B. Jana , A. Ghosh , S. Maiti , D. Bain , S. Banerjee , H. N. Ghosh , A. Patra , J. Phys. Chem. C 2016, 120, 25142–25150.

[194] B. M. Graff , B. P. Bloom , E. Wierzbinski , D. H. Waldeck , J. Am. Chem. Soc. 2016, 138, 13260–13270. 27636121 10.1021/jacs.6b06991

[195] B. Valeur , M. N. Berberan‐Santos , in Molecular Fluorescence: Principles and Applications, Wiley‐VCH Verlag GmbH & Co. KGaA, Weinheim, Germany 2012, pp. 213–261.

[196] S. Melle , O. G. Calderón , M. Laurenti , D. Mendez‐Gonzalez , A. Egatz‐Gómez , E. López‐Cabarcos , E. Cabrera‐Granado , E. Díaz , J. Rubio‐Retama , J. Phys. Chem. C 2018, 122, 18751–18758. 10.1039/c9nr02039j 31294740

[197] Z. Li , Y. Zhang , S. Jiang , Adv. Mater. 2008, 20, 4765–4769.

[198] T.‐L. Nguyen , P. Spizzirri , G. Wilson , P. Mulvaney , Chem. Commun. 2009, 9, 174–176. 10.1039/b814771j 19099059

[199] C. Yan , A. Dadvand , F. Rosei , D. F. Perepichka , J. Am. Chem. Soc. 2010, 132, 8868–8869. 20536139 10.1021/ja103743t

[200] A. Bednarkiewicz , M. Nyk , M. Samoc , W. Strek , J. Phys. Chem. C 2010, 114, 17535–17541.

[201] S. Alyatkin , I. Asharchuk , K. Khaydukov , A. Nechaev , O. Lebedev , Y. Vainer , V. Semchishen , E. Khaydukov , Nanotechnology 2017, 28, 035401. 27928995 10.1088/1361-6528/28/3/035401

[202] A. M. Kotulska , A. Pilch‐Wróbel , S. Lahtinen , T. Soukka , A. Bednarkiewicz , Light Sci. Appl. 2022, 11, 256. 35986019 10.1038/s41377-022-00946-x PMC9391450

[203] A. Pilch‐Wrobel , A. M. Kotulska , S. Lahtinen , T. Soukka , A. Bednarkiewicz , Small 2022, 18, 2200464. 10.1002/smll.202200464 35355389

[204] R. Marin , L. Labrador‐Paéz , A. Skripka , P. Haro‐González , A. Benayas , P. Canton , D. Jaque , F. Vetrone , ACS Photonics 2018, 5, 2261–2270.

[205] M. Zeng , S. Singh , Z. Hens , J. Liu , F. Artizzu , R. V. Deun , J. Mater. Chem. C 2019, 7, 2014–2021.

[206] L. Ruan , Y. Zhang , ACS Appl. Mater. Interfaces 2021, 13, 51362–51372. 34664937 10.1021/acsami.1c14711

[207] W. Zhang , J. Li , H. Lei , B. Li , Opt. Express 2020, 28, 12450. 32403742 10.1364/OE.386601

[208] L. Francés‐Soriano , S. Gonzalez‐Carrero , E. Navarro‐Raga , R. E. Galian , M. González‐Béjar , J. Pérez‐Prieto , Adv. Funct. Mater. 2016, 26, 5131–5138.

[209] S. Kim , D. H. Shin , J. Kim , C. W. Jang , S. S. Kang , J. M. Kim , J. H. Kim , D. H. Lee , J. H. Kim , S.‐H. Choi , S. W. Hwang , Sci. Rep. 2016, 6, 27145. 27250343 10.1038/srep27145 PMC4889998

[210] S. Kundu , S. Bhattacharyya , A. Patra , Mater. Horiz. 2014, 2, 60–67.

[211] S. Jana , X. Xu , A. Klymchenko , A. Reisch , T. Pons , ACS Nano 2021, 15, 1445–1453. 33378154 10.1021/acsnano.0c08772

[212] A. Gopi , M. Sajitha , R. Haridas , L. Varghese , K. Yoosaf , Chem. Asian J. 2018, 13, 1492–1499. 29573188 10.1002/asia.201800328

